# XROMM Analysis of Feeding Mechanics in Toads: Interactions of the Tongue, Hyoid, and Pectoral Girdle

**DOI:** 10.1093/iob/obac045

**Published:** 2022-11-15

**Authors:** R M Keeffe, R W Blob, D C Blackburn, C J Mayerl

**Affiliations:** Department of Biological Sciences, Mount Holyoke College, South Hadley, MA 01075, USA; Department of Biological Sciences, Clemson University, Clemson, SC 29634, USA; Department of Biology, University of Florida, Gainesville, FL 32611, USA; Florida Museum of Natural History, University of Florida, Gainesville, FL 32611, USA; Department of Biological Sciences, Northern Arizona University, Flagstaff, AZ 86011, USA

## Abstract

During feeding in many terrestrial vertebrates, the tongue acts in concert with the hyoid and pectoral girdle. In frogs, these three elements are interconnected by musculature. While the feeding mechanics of the anuran tongue are well-studied, little is known of how the motions of the tongue relate to the movements of the skeleton or how buccal structures move following closure of the mouth. Although features such as the pectoral girdle and hyoid are not externally visible in frogs, their motions can be tracked in X-ray video. We used XROMM (X-ray Reconstruction of Moving Morphology) techniques to track the 3D movements of the tongue, hyoid apparatus, pectoral girdle, skull, and jaw during the feeding cycle of the cane toad, *Rhinella marina*. We show how the movements of these elements are integrated during tongue protrusion and prey capture, as well as during prey transport, swallowing, and recovery. Our findings suggest that the hyoid apparatus is important both for prey manipulation and swallowing. The tongue consistently stretches posterior to the skull during swallowing, often more than it stretches during protrusion to reach the prey. Feeding kinematics are similar between individuals, and the kinematics of unsuccessful strikes generally resemble those of successful strikes. Our data also provide a new perspective on the potential role of the pectoral girdle, an element with a predominant locomotor function, during feeding events. This work raises new questions about the evolution of feeding in frogs, as well as how the diversity of pectoral and buccal anatomy observed across anurans may influence feeding kinematics.

## Spanish

Durante la alimentación en muchos vertebrados terrestres, la lengua actua en combinación con el hioides y la cintura ó faja pectoral. En las ranas, estos tres elementos estan conectados con músculos. Aunque la mecánica de la alimentación de la lengua de los anuros esta bien estudiada, se sabe poco acerca de como el movimiento de la lengua se relaciona con los movimientos del esqueleto or como se mueven as estructuras bucales despues de que se cierra la boca. Mientras que algunas características como la cintura pectoral y el hioides no se ven externamente en las ranas, sus movientos se pueden seguir usando video de rayos X. Usamos técnicas de XROMM (reconstrucción de rayos x de morfología en movimiento) para seguir los movimientos de la lengua, el hyodeo, la cintura pectoral, cráneo y mandibula en 3D, durante el ciclo de alimentación del sapo de caña *Rhinella marina*. Mostramos que los movimientos de estos elementos estan integrados durante la protrusión de la lengua y la captura de la presa, ademas de durante el transporte de la presa, tragado y recuperación. Nuestros resultados demuestran que el hioides es importante para manipular la presa y para tragar. La lengua se estira posterior al cráneo durante la fase de tragar, muchas veces aún más que el estiramiento que presenta cuando atrapa la presa. La cinemática de la alimentación es similar entre individuos y la cinemática de intentos fallidos es similar a la de los intentos exitosos. Nuestros datos proven una nueva perspectiva del rol potential en la alimentación de la cintura pectoral, un element que se usa primordialmente en la locomoción. Este trabajo sugiere nuevas preguntas acerca de la evolución de la alimentación en las ranas, así como de la influencia de la diversidad de los pectorales y la anatomía buccal que se observan en los anuros en la cinemática de alimentación.

## Introduction

Feeding in vertebrates is an exemplar system for functional morphologists to understand how a complex behavior relates to organismal fitness and evolutionary trajectories (Liem and Osse 1975; Ungar 2010; Anderson et al. 2011; Wainwright et al. 2012). Feeding in frogs (order Anura) is of particular interest due to its dramatic, ballistic nature (Gans and Gorniak 1982a; Lappin et al. 2006). In most frogs, the tongue is attached in the front of the mouth and moves forward under its own inertia as it is flipped out of the mouth to catch prey, often undergoing incredible acceleration (>310 m/s^2^; Nishikawa and Gans 1996). The mechanism of the anuran “tongue flip” has been explored by biologists since the 1800s (see Gans and Gorniak 1982a). Over the past ∼40 years, advancements in experimental techniques such as EMG (electromyography), high speed video, and XROMM (X-ray Reconstruction of Moving Morphology) have led to many new insights in anuran feeding mechanisms (Emerson 1977; Gans and Gorniak 1982a; Nishikawa 2000; Witzmann et al. 2019).

Recent work on anuran feeding biomechanics has underscored remaining gaps in understanding due to limitations of the tools used in previous studies (Nishikawa 2000). One area in which uncertainty has persisted is in the function of the hyoid apparatus (Emerson 1977; Gans and Gorniak 1982a, 1982b). In her review of feeding in frogs, Nishikawa (2000) points out several reasons for the difficulty in determining the mechanisms in this system. First, feeding motions are incredibly fast and involve the simultaneous activation of many different muscles. This makes determining muscle order and function difficult even with EMG data (Nishikawa and Gans 1996). Second, interpretations of muscle stimulation experiments are sometimes undermined by tonic muscle contractions that oppose normal movements. For example, tonic contractions of m. levator mandibulae resist mouth opening even when the m. depressor mandibulae is stimulated in *R. marina* (Nishikawa and Gans 1992; Nishikawa 2000). To address challenges like these, Nishikawa (2000) advocated for the use of diverse kinematic and anatomical techniques to resolve questions about anuran feeding.

Beyond the complexities of tongue protrusion in frogs, even less is known about the functional role of these structures during the prey transport and swallowing phases of a feeding cycle. Previous work with EMG and high-speed video focused on lingual protrusion (Gans and Gorniak 1982a; Matsushima et al. 1985; Nishikawa and Roth 1991; Deban and Nishikawa 1992; Nishikawa and Gans 1996; Nishikawa et al. 1999; Wolff et al. 2004; Lappin et al. 2006; Monroy and Nishikawa 2009), with limited examination of functional mechanisms once the frog's mouth is closed. However, prey transport and swallowing phases of the feeding cycle are known to be complex and important in vertebrates such as mammals (Smith 1992; Mayerl et al. 2021). Several studies on frog feeding mechanics have remarked on the swallowing mechanism (Regal and Gans 1976; Ritter and Nishikawa 1995; Yoshida 2001), but few have examined it specifically (Levine et al. 2004; Witzmann et al. 2019). Some have hypothesized that the tongue is the main functional organ for swallowing prey (Regal and Gans 1976; Duellman and Trueb 1986; Gray et al. 1997; Nishikawa 2000) and posited that the hyoglossus muscle and tongue contribute to prey transport and swallowing (Ritter and Nishikawa 1995; Tso et al. 1995). However, EMG-based studies of the buccal musculature during feeding did not include data during the swallowing phase (Gans and Gorniak 1982a). The role of hyoid movement has also not typically been discussed in detail (although see Emerson 1977), despite the complexity of muscles associated with it in the buccal cavity (Ecker 1889) and it being known to be involved in swallowing in other vertebrates (Liem 1973; Crompton et al. 1975; Lombard and Wake 1976; Mayerl et al. 2021).

In this paper, we bridge these gaps in knowledge of the feeding cycle in frogs. Our goal is to define the movements of the hyoid apparatus, tongue, and pectoral girdle during feeding and swallowing, including the relative timings of their movements and their functional roles during swallowing. We also aim to examine variation across individuals and strikes of different performance to evaluate the stereotypy of this ballistic behavior. To accomplish these goals, we used XROMM (Brainerd et al. 2010) to visualize structures throughout the feeding cycle of the cane toad, *Rhinella marina*, including after the mouth was closed. *Rhinella marina* is a common model in studies of frog feeding (Emerson 1977; Gans and Gorniak 1982a, 1982b; Nishikawa and Gans 1996) and was particularly appropriate for this study because the large body sizes they achieve help to simplify the implantation of markers for XROMM methods, and their typically voracious feeding enabled the collection of multiple trials under experimental conditions (Zug and Zug 1979).

## Methods

### Animal husbandry

Three large adult cane toads were purchased from a commercial supplier (Gator City Reptiles, Gainesville, FL). Because body size can influence the feeding kinematics of bufonids (O'Reilly et al. 1995), we chose toads that were similar in body length (RM01 = 23.80 cm SVL; RM02 = 19.59 cm SVL; RM03 = 20.32 cm SVL). Toad care followed standard amphibian husbandry procedures (Pough 2007) and all animal care and procedures complied with UF IACUC protocol #202011027 and NEOMED IACUC protocol #20-12-287.

### Surgical procedures

Each toad was anesthetized with dilute buffered tricaine methanesulfonate (MS-222) (Tricaine methanesulfonate, MilliporeSigma, USA; 2 g/L) prior to surgical implantation of 0.8 mm tantalum markers (X-Medics, Frederiksberg, Denmark) into the pectoral girdle, tongue, and hyoid apparatus (Gentz 2007). Butorphanol, an analgesic, was administered by intramuscular injection (0.3 mg/kg) into the muscle of the right arm following successful induction (Gentz 2007).

**Fig. 1:**
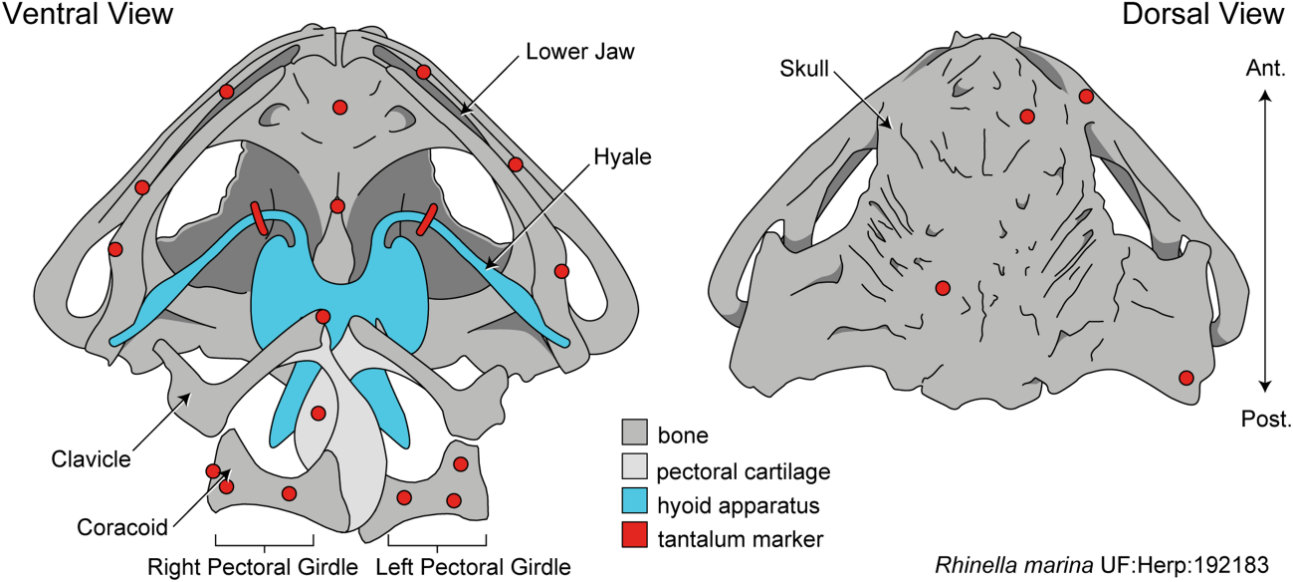
The skull of *R.marina* in ventral and dorsal view showing the locations of the X-ray markers, indicated in red. Hyoid apparatus indicated in blue, pectoral cartilage in light gray, and bone in darker gray.

We used aseptic surgical methods to associate eight markers with the pectoral girdle. Because toads have an arciferal girdle type (i.e., the right and left halves are not fused at the midline; Emerson 1984), markers were needed on both sides to measure intragirdle movements (see Fig. 1 for an illustration of marker positions). During surgery, a ventral incision was made from left to right at the level of the pectoral girdle. We used a probe to palpate connective tissue at the sternum to locate the distal end of the coracoid, and accessed bone by spreading the pectoral musculature. A handheld pin vise with a 0.8 mm diameter bit was used to drill two shallow holes into the distal end of the coracoid. For each of these two locations, sterile petroleum jelly held the tantalum marker to the end of the probe until it was pressed into the drilled hole and positioned to be flush with the surface of the bone. After placing the distal markers, we parted the muscle bodies along the shaft of the coracoid until its midshaft region was exposed, after which we drilled an additional 0.8 mm hole and placed a tantalum bead into the midshaft location. We repeated these procedures on the other side of the girdle. We then sutured laser-drilled 1.00 mm markers to the posterior and anterior margins of the pectoral girdle through the connective tissue tightly associated with the midline pectoral cartilages. The ventral incision was then sutured closed.

**Fig. 2:**
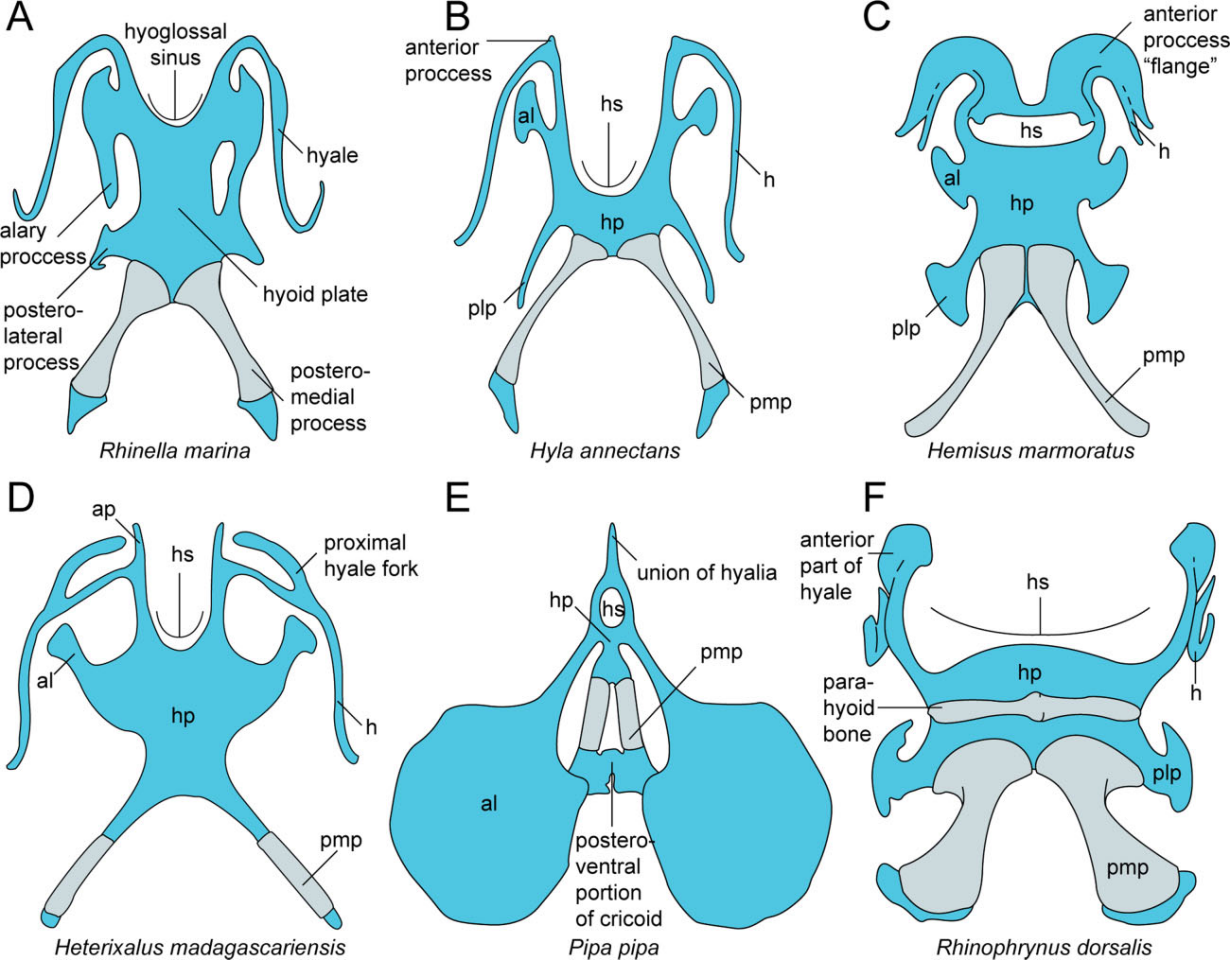
An illustration of various anuran hyoid morphologies in ventral view, cartilaginous regions are blue and the posteromedial and parahyoid bones are light gray. Species depicted are as follows: (**A**) *R.marina*, (**B**) *Hyla annectans*, (**C**) *Hemisus marmoratus*, (**D**) *Heterixalus madagascariensis*, (**E**) *Pipa pipa*, and (**F**) *Rhinophrynus dorsalis*. Abbreviations: al = alary process; ap = anterior process; h = hyale; hp = hyoid plate; hs = hyoglossal sinus; plp = posterolateral process; pm = posteromedial process. A–D adapted from Trewavas 1932; E from Ridewood 1897; and F from Duellman and Trueb 1986. The asymmetry of *R.marina* is not typical for the species.

After incision closure we attached several additional markers to structures of the mouth, hyoid, and skull. We used a spinal needle plunger to insert two 0.8 mm tantalum markers into the tongue, one at the base and one at the distal tip. With the mouth held open, we attached one X-ray dense nerve clip (Weck Hemoclip Traditional Ligating Clips, Teleflex Inc., Research Triangle Park, NC) onto the anterior loops of both the right and left hyalia, roughly in the center of each hyale (see Fig. 2A for illustration of *R. marina* hyoid anatomy). Anterior margins of the hyale are accessible superficially as they are embedded within the floor of the mouth. As toads have relatively dry skin, we were able to attach four tantalum markers with cyanoacrylate glue to the skin of the skull and six onto the skin of the lower jaw. In *R. marina*, the skin is tightly associated with the skull and jaw, allowing such surface markers to be used to accurately reflect the motions of these structures. Between 20 and 22 (two additional markers were placed into the tongue of RM02) markers were associated with each individual toad in total.

### Data collection

We collected video data of the toads feeding using high-speed videofluoroscopy (GE 9400 C-Arm,80 kvP, 6.6 mA) coupled with a high-speed video camera (250 FPS, XC1M digital camera, XCitex, Cambridge, MA) following standard XROMM undistortion and calibration procedures (Brainerd et al. 2010). Cane toads were placed into a Plexiglas enclosure positioned between two C-arms and high-speed cameras to collect light and X-ray video. Prey (crickets; *Gryllodes sigillatus*) were lightly dusted with barium (E-Z Paque Barium Sulfate, EZ EM Inc., NY) so they could be better seen on the X-ray videos. Prey were then dropped into the enclosure in front of the toads using metal tongs. Events were marked in the recording periods with a trigger marker and then cropped after the recording session. Bouts of trials occurred in 15-to-20-min intervals. We collected 50 to 100 feeding strikes per individual. Following video data collection, the toads were euthanized via immersion in buffered MS-222 (6 g/L) and then frozen. After the animals were frozen, we completed a precision trial (Brainerd et al. 2010; Menegaz et al. 2015) with one of the toads to determine what amount of marker movement could be attributed to noise. At the end of data collection, toads were accessioned into the FLMNH herpetology collection (UF-Herp-192183, RM01; UF-Herp-193038, RM02; UF-Herp-192184, RM03).

Computed tomographic (CT) and diffusible iodine-based contrast-enhanced CT (diceCT) data were collected at the University of Florida's Nanoscale Research Facility on a GE phoenix v|tome|x m scanner. CT scans of RM01 had a voxel size of 0.08710214 mm, RM02 had a voxel size of 0.625 mm, and RM03 had a voxel size of 0.06491147 mm. To prepare the specimen for diceCT, UF-Herp-192184 (RM03) was stained in 1.25% I_2_Kl for 5 months, with the staining solution refreshed every few weeks. Visualizations and segmentation of the anatomical elements were performed in VGSTUDIO MAX v.3.2 (Volume Graphics, Heidelberg, Germany).

We also collected anatomical data via dissection of museum specimens to verify our interpretations of the internal anatomy movements we hypothesized from the X-ray video data. Three individuals of *R. marina* (UF-Herp-192557, UF-Herp-192558, UF-Herp-192559) were collected from Jupiter, FL and then transported to FLMNH and euthanized in buffered MS-222 (6 g/L). Sagittal and lateral incisions were made to the gular region to expose the hyoid apparatus, and the hyoid was then manipulated into various states of extension. Measurements were taken with digital calipers and a piece of twine, and the resulting positions were recorded (Supp. Table 1).

### Data processing

From the collected X-ray videos of feeding events, we chose 13 to 16 events per toad that spanned the full range of prey distances for analysis, 44 events total. We included examples of three infrequently observed feeding events: a miss, a sideways strike, and a double-swallow. A miss occurs when a toad attempts to catch the prey and fails to do so (Video 1; Supp. Fig. 1). A sideways strike occurs when the toad rapidly rotates its head toward its left or its right to catch the prey (Video 2; Supp. Fig. 2), rather than simply leaning forward. A double-swallow occurs when the toad attempts to push the prey into the esophagus more than once (Video 3; Supp. Fig. 3) or has a delayed swallow (Video 4; Supp. Fig. 4). The X-ray videos for these 44 events were each loaded into XMALab v.1.5.5 (Knörlein et al. 2016) for distortion correction, calibration, marker tracking, and calculating the movements of the rigid bodies of the skull, lower jaw, and both right and left halves of the pectoral girdle. Mean marker-tracking precision was 0.008593 cm ± 0.009048 cm (mean ± standard deviation, *n* = 1251 distances between two markers within the skull rigid body).

The positions of each tantalum marker were tracked from 10 frames before first head movement to the last frame following a full throat pump after the tongue returned to resting position following a swallow. We consider the first frame of head movement to mark the beginning of a feeding cycle. The last frame of a feeding cycle is when the tongue returns to its resting length in the buccal cavity. From these definitions we calculated the number of frames per feeding event. We also recorded the frames on which other kinematic events occurred (such as eye closure, tongue protrusion, tongue retraction, mouth opening, etc., see Supp. Table 2 for full list and Supp. Table 3 for column definitions). From those data we calculated the relative timings and durations per event (Tables 1–3; Supp. Table 4).

**Table 1:** Relative timing of kinematic variables for individuals RM01, RM02, and RM03 as well as the average between all three individuals. Values written mean ± SE for successful, single-swallow, straight events for each individual (RM01 = 11; RM02 = 13; RM03 = 12). See Supp. Table 4 for definitions of variables.

	RM01	RM02	RM03	Overall
Prey Capture Phase				
Eye Closure Onset (cycle %)	0	0	0	0
Mouth Open Onset (cycle %)	9.51 ± 0.15	7.9 ± 0.21	8.33 ± 0.19	8.54 ± 0.16
Hyalia Max1 (cycle %)	9.49 ± 0.14	8.62 ± 0.19	8.85 ± 0.15	8.96 ± 0.11
Tongue Base Protrusion Onset (cycle %)	9.56 ± 0.18	8.94 ± 0.16	8.7 ± 0.16	9.05 ± 0.11
Tongue Tip Protrusion Onset (cycle %)	9.62 ± 0.24	9.51 ± 0.17	9.31 ± 0.16	9.48 ± 0.11
Hyalia Min1 (cycle %)	10.25 ± 0.16	9.96 ± 0.16	9.79 ± 0.17	9.99 ± 0.1
Hyalia Max2 (cycle %)	11.53 ± 0.16	11.37 ± 0.26	10.77 ± 0.13	11.22 ± 0.12
Tongue Tip Protrusion Offset (cycle %)	13.19 ± 0.26	12.16 ± 0.22	12.25 ± 0.18	12.51 ± 0.15
Tongue Base Protrusion Offset (cycle %)	13.1 ± 0.25	13.08 ± 0.23	12.41 ± 0.24	12.86 ± 0.14
Prey Transport Phase				
Maximum Mouth Gape (cycle %)	16.51 ± 0.3	16.94 ± 0.33	15.92 ± 0.21	16.47 ± 0.18
Tongue Base Retraction Offset (cycle %)	19.33 ± 0.43	21.02 ± 0.56	20.33 ± 0.19	20.27 ± 0.27
Tongue Tip Retraction Offset (cycle %)	20.5 ± 0.54	19.96 ± 0.53	25.07 ± 0.65	21.83 ± 0.5
Skull Max Stretch (cycle %)	15.76 ± 0.44	28.45 ± 2.83	24.68 ± 1	23.32 ± 1.38
Mouth Open Offset (cycle %)	22.08 ± 0.54	24.62 ± 0.55	23.98 ± 0.59	23.63 ± 0.36
Eye Closure Offset (cycle %)	20.68 ± 0.84	32.41 ± 2.43	32.22 ± 1.76	28.76 ± 1.39
Hyalia Min2 (cycle %)	27.61 ± 0.49	32.36 ± 0.77	27.34 ± 0.82	29.23 ± 0.57
Girdle Max Yaw (cycle %)	28.69 ± 0.66	30.73 ± 2.82	N/A	29.79 ± 1.54
Tongue Base Max2 (cycle %)	30.51 ± 0.43	N/A	N/A	30.51 ± 0.43
Hyoid Dorsal Maximum (cycle %)	30.51 ± 0.4	36.07 ± 0.79	34.54 ± 0.98	33.86 ± 0.59
Hyalia Max3 (cycle %)	31.39 ± 0.46	38.21 ± 1.09	35.33 ± 0.89	35.16 ± 0.69
Swallowing Phase				
Hyalia Max4 (cycle %)	60.86 ± 0.97	52.77 ± 1.05	N/A	56.48 ± 1.1

**Table 2:** Event durations in milliseconds (ms) of kinematic variables for individuals RM01, RM02, and RM03 as well as the average between all three individuals. Values written mean ± SE for successful, single-swallow, straight events for each individual (RM01 = 11; RM02 = 13; RM03 = 12). See Supp. Table 4 for definitions of variables.

	RM01	RM02	RM03	Overall
Cycle Duration (ms)	1537.82 ± 51.08	1491.38 ± 21.96	1546 ± 20.69	1523.78 ± 18.7
Prep. phase (ms)	144.73 ± 4.85	116.31 ± 2.54	127.33 ± 2.78	128.67 ± 2.74
Eye Closure Duration (ms)	312.73 ± 12.08	474.15 ± 29.95	493 ± 26.88	431.11 ± 19.43
Mouth Open Duration (ms)	189.82 ± 7.46	246.15 ± 5.12	240.33 ± 11.49	227 ± 6.28
Tongue Tip Protrusion Duration (ms)	53.82 ± 4.89	39.08 ± 1.03	45 ± 1.11	45.56 ± 1.84
Tongue Tip Retraction Duration (ms)	106.55 ± 5.59	110.77 ± 5.66	191.67 ± 8.7	136.44 ± 7.63
Tongue Base Protrusion Duration (ms)	53.09 ± 0.95	60.92 ± 1.03	56.67 ± 2.19	57.11 ± 1
Tongue Base Retraction Duration (ms)	93.82 ± 1.25	116.62 ± 5.58	121.33 ± 4.07	111.22 ± 3.11
Hyalia Spike 1 Duration (ms)	19.27 ± 0.73	20.62 ± 2.39	15 ± 1.31	18.33 ± 1.05
Hyoid Dorsal Scrape Duration (ms)	57.82 ± 5.51	85.54 ± 6.22	122 ± 3.17	89.22 ± 5.24

**Table 3:** Quantitative measurements of kinematic variables for individuals RM01, RM02, and RM03 as well as the average between all three individuals. Values written mean ± SE for successful, single-swallow, straight events for each individual (RM01 = 11; RM02 = 13; RM03 = 12). See Supp. Table 4 for definitions of variables.

	RM01	RM02	RM03	Overall
Max Gape Angle (degrees)	67.79 ± 0.78	63.69 ± 0.75	78 ± 1.31	69.71 ± 1.17
Tongue Rest (cm)	1.94 ± 0.06	2.55 ± 0.03	2.48 ± 0.06	2.34 ± 0.05
Max Tongue Protrusion Stretch (cm)	4.49 ± 0.19	3.89 ± 0.16	4.1 ± 0.19	4.14 ± 0.11
Max Tongue Protrusion Stretch/Head Length	0.83 ± 0.03	0.79 ± 0.03	0.8 ± 0.04	0.81 ± 0.02
Max Tongue Protrusion Stretch/Rest Length	2.32 ± 0.09	1.53 ± 0.06	1.66 ± 0.08	1.81 ± 0.07
Max Tongue Retraction Stretch (cm)	4.69 ± 0.05	3.98 ± 0.04	4.96 ± 0.08	4.52 ± 0.08
Max Tongue Retraction Stretch/Head Length	0.86 ± 0.01	0.81 ± 0.01	0.97 ± 0.02	0.88 ± 0.01
Max Tongue Retraction Stretch/Rest Length	2.43 ± 0.06	1.56 ± 0.02	2.01 ± 0.05	1.98 ± 0.07
Hyalia Rest (cm)	2.41 ± 0.02	2.67 ± 0	2.81 ± 0.01	2.64 ± 0.03
Hyalia Min (cm)	1.84 ± 0.04	2.42 ± 0.02	1.25 ± 0.03	1.85 ± 0.08
Hyalia Max (cm)	3.03 ± 0.02	2.78 ± 0.01	2.85 ± 0.02	2.89 ± 0.03
Girdle Max Yaw (degrees)	19.74 ± 2	14.43 ± 2.3	N/A	16.86 ± 1.61
Skull Max Stretch (cm)	1.49 ± 0.09	0.54 ± 0.02	0.84 ± 0.04	0.93 ± 0.07
SVL (cm)	23.8	19.59	20.32	21.24 ± 1.3
Head Length (cm)	5.43	4.94	5.1	5.16 ± 0.14

We exported CSV files of the rigid body transformations of the skull, jaw, right and left pectoral girdle, as well as the three-dimensional marker positions. Additionally, we exported the intermarker distances between the two tongue markers, the two hyoid markers, the distal-most markers of the right and left halves of the pectoral girdle, and a skull and midline girdle marker from XMALab as CSV files. These data were then used to calculate additional kinematic variables (Table 3, Supp. Table 2). We concatenated all event files based on individual and chronological event order, so that kinematics could be processed in the same Maya animation.

We imported STL files into Autodesk Maya (2022, Autodesk Inc., San Rafael, CA) that were generated from the segmented CT data of each toad and cleaned in Meshlab (Cignoni et al. 2008). In Maya, we generated an anatomical coordinate system (ACS) using three planes oriented to generate a single point of intersection between them to standardize starting position across all animals (Brainerd et al. 2010; Mayerl et al. 2016). For each individual, we aligned the skull and lower jaw with the ACS such that this point of intersection was positioned equidistant between the right and left margins of the skull, at the anteroposterior position where the pterygoid makes contact with the squamosal. This positions the ACS with the transverse, sagittal, and frontal planes relative to the skull. From this orientation, we created a joint coordinate system (JCS) such that the X-axis pointed anteriorly and represented anteroposterior translations and roll rotations, the Y-axis measured mediolateral movements, with positive rotations indicating ventral pitching movements, and the Z-axis measured dorsoventral translations (positive values indicating dorsal movements), rotations indicating yawing rotations of the skull. We duplicated this system and parented one JCS to the skull and the other to the jaw. We then moved the right half of the pectoral girdle so that (1) the centroid of the STL was positioned underneath the right half of the skull, (2) the medial margin of the coracoid intersected the point of intersection of the ACS, (3) the transverse plane bisected the coracoid anteroposteriorly, and (4) the frontal plane bisected the coracoid dorsoventrally. We did the same for the left half of the pectoral girdle, mirrored from the right half of the pectoral girdle across the sagittal plane. The JCS to measure girdle movements was oriented the same way as the skull and jaw JCSs, except that its point of intersection was translated to the right of the ACS point of intersection to overlap with the center of the glenoid fossa on either side of the pectoral girdle. Each girdle JCS was parented to its respective girdle. This positioning of the ACS, rigid bodies, and JCSs creates a standardized reference pose for all three individuals from which translations and rotations during feeding behaviors can be measured (see Supp. Fig. 5 for illustrations of the ACS and JCS positions). Each JCS was parented to its respective rigid body so that they would translate with the rigid bodies during Maya animations.

We then imported the translations and rotations of those bones from corresponding data output from XMALab to animate bones following standard XROMM protocols (Brainerd et al. 2010) using the XROMM toolshelf (xrommwiki.org). We also imported the translations of the individual markers for each trial. Translations of the two tongue markers and right hyoid marker were measured relative to the posterior margin of the lower jaw throughout all feeding events per toad and exported as CSV files. Other exported measures included translations and rotations of the skull relative to the pectoral girdle, the right pectoral girdle relative to the left pectoral girdle, and the jaw relative to the skull.

Illustrations of the soft and hard tissue movements were produced in Adobe Illustrator (version 26.1; Adobe Systems, San Jose, CA), using a combination of references including X-ray video frames, Maya animations, dissection photos, CT scans, diceCT scans, and distance data.

### Statistical analyses

We imported kinematic output from Maya into RStudio (version 1.4.1717; RCT 2021) where the frames for each feeding event were isolated and aligned for all kinematic variables. These cropped data were spline interpolated to 101 evenly spaced increments to standardize the durations of the feeding events. Average kinematic profiles (with standard deviations) were calculated from those splined data for the successful, single-swallow, straight strikes (RM01, *n* = 11; RM02, *n* = 13; RM03, *n* = 12) and plotted in R (package ggplot; Wickham 2016). Double-swallows, side strikes, and misses were excluded from these averages and plotted separately. To quantify the kinematic variation between our three individuals and between successful and atypical feeding events (double-swallows, misses, side strikes), we performed a Pearson's correlation analysis (*cor* function; package stats, R version 3.6.2) for each kinematic variable (Supp. Table 5). The results from these correlation analyses were then summarized (Table 4) and values over 0.7 were highlighted for a heuristic illustration of strong correlations.

**Table 4:** A summary of the results from our Pearson's correlation tables (see Supp. Table 5 for full matrices). Columns 1–4 indicate the kinematic variable compared. Column 5 is the average of three correlation coefficients of the average kinematics between successful, single-swallow, straight events between the three individuals (RM01: RM02; RM01: RM03; RM02: RM03). All remaining columns are average correlation coefficients for the titular atypical event type against the average kinematics of successful, single-swallow, straight events for the same individual. Cells are color coded by the value of the correlation coefficient, with darker colors indicating stronger correlations. Red colors indicate relatively low values and blue colors indicate relatively high values. Correlation values over 0.7 are in bold.

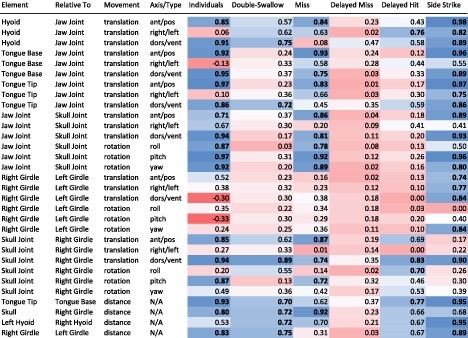

We tabulated our timing variables (e.g., maximum tongue protrusion, maximum jaw gape angle; see Supp. Table 2) for each event and converted the frame counts into duration in seconds by dividing the frame number by the frames per second (FPS) setting of our X-ray camera (250 FPS). These timing data were converted into cycle percentages by multiplying the seconds value by 101 and then dividing by the cycle duration for each event. We then calculated the mean and standard error (SE) for each of these variables for each individual for: successful, single-swallow, straight events (RM01, n = 11; RM02, *n* = 13; RM03, *n* = 12), miss events (RM01, *n* = 1; RM03, *n* = 1), double-swallow events (RM01, *n* = 1; RM03, *n* = 1), side strike events (RM01, *n* = 1; RM03, *n* = 1), delayed-swallow misses (RM03, *n* = 1), and successful, delayed-swallow events (RM01, *n* = 1).

**Fig. 3:**
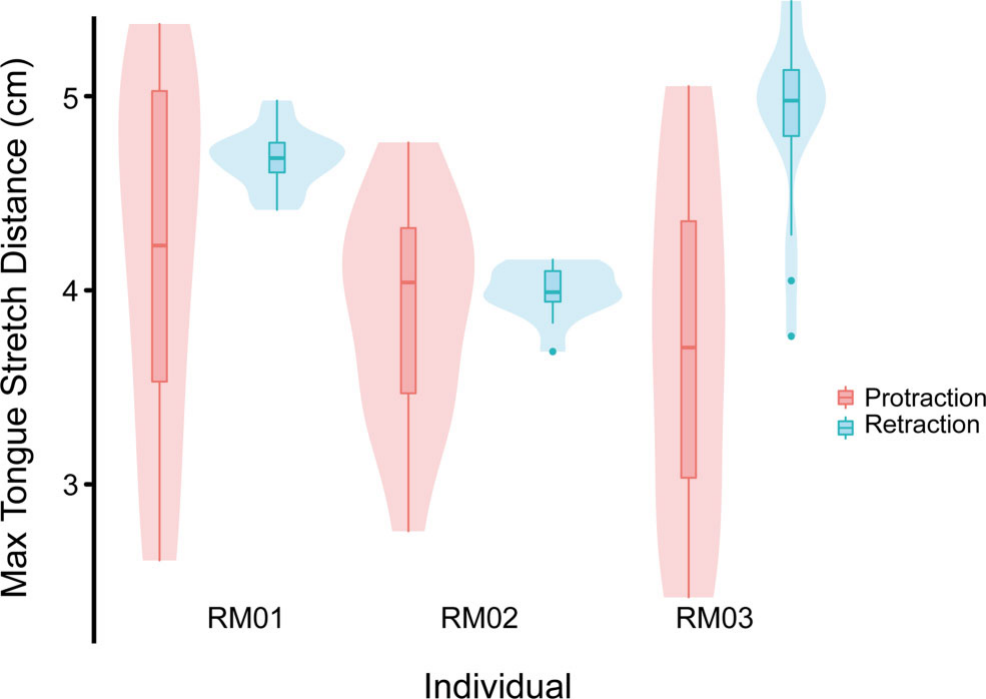
Box plots showing maximum distance between the tongue base and tongue tip markers during tongue protrusion (pink plots) and retraction (blue plots). The middle line of each box plot represents the median, the upper and lower hinges represent the first and third quartiles, and the upper whiskers extend to 1.5 times the interquartile range from their respective hinge. Data beyond the whiskers are plotted as individual points. The first two plots from the left are those of all recorded feeding events for RM01 (*n* = 15), the middle two plots are those for all feeding events of RM02 (*n* = 13), and farthest two plots on the right are those of RM03 (*n* = 16). All box plots are superimposed over violin plots of their same data. The violin plots visualize the distribution of the data points along the Y-axis.

All additional analyses were conducted in R. We used box plots, superimposed over violin plots, to show the maximum distances between the two tongue markers during retraction and protraction (package ggplot; Wickham 2016; Fig. 3). We assessed differences in variance between these groups with a series of Levene's tests (*leveneTest* function; package car) and compared the means between groups with Welch two-sample *t*-tests (*t.test* function; package stats; Supp. Table 6). We calculated several linear models (*lm* function; package stats) to assess the relationship between certain timing and distance variables: mouth opening onset and tongue tip protrusion onset; moment of minimum tongue intermarker distance and moment of maximum gape; and tongue tip protrusion offset and maximum tongue protrusion distance (Supp. Table 6). We also conducted a linear mixed effect model (*lmer* function; package lme4; Bates et al. 2015) and an ANOVA (*Anova* function; package car) to assess the role of variables such as hits vs. misses and single vs. double swallows, with individual being randomized (Supp. Table 6). The results of this ANOVA should be treated with caution given the necessary small sample size, however. We extracted the maximum jaw gape angle and cycle timing variables for each event and ran a principal component analysis (PCA) from these data (*prcomp* function; package stats) and plotted the first two PC axes (*autoplot* function; package ggfortify) to better visualize the multivariate space of our dataset (Tang et al. 2016; Horikoshi and Tang 2018).

## Results

### Feeding cycle event timing

**Fig. 4:**
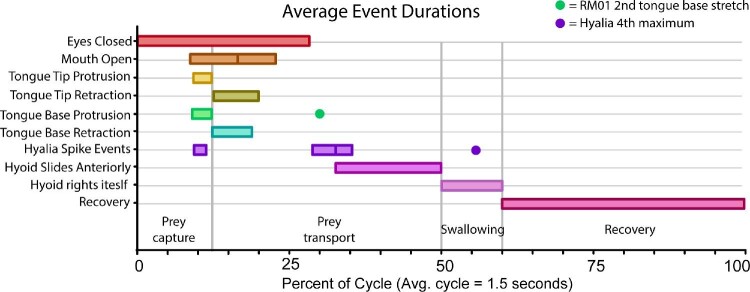
Relative durations of major kinematic events for individual RM01 as a percentage of a full feeding cycle (see Table 1). Horizontal gray lines delimitate the different kinematic variables, vertical gray lines indicate transitions between the cycle phases of prey capture, prey transport, swallowing, and recovery. The lines within event duration bars are maximum jaw gape for Mouth Open and hyoid dorsal maximum for the second hyalia spike event. The green dot for Tongue Base Protrusion indicates the second, minor stretching event of the tongue base marker that occurs concurrently with the hyoid dorsal maximum only for individual RM01. The purple dot indicates the time at which the fourth hyalia intermarker maximum occurs.

Across the 36 normal feeding trials analyzed, we found that the average length of a full feeding cycle—from the beginning of head movement to the moment the tongue returns to a resting length in the mouth—is 1.52 ± 0.28 s (mean ± SE; Table 2). The feeding cycle can be broken down into four major phases: prey capture, prey transport, swallowing, and recovery (Fig. 4; Table 1).

#### Prey capture phase

**Fig. 5:**
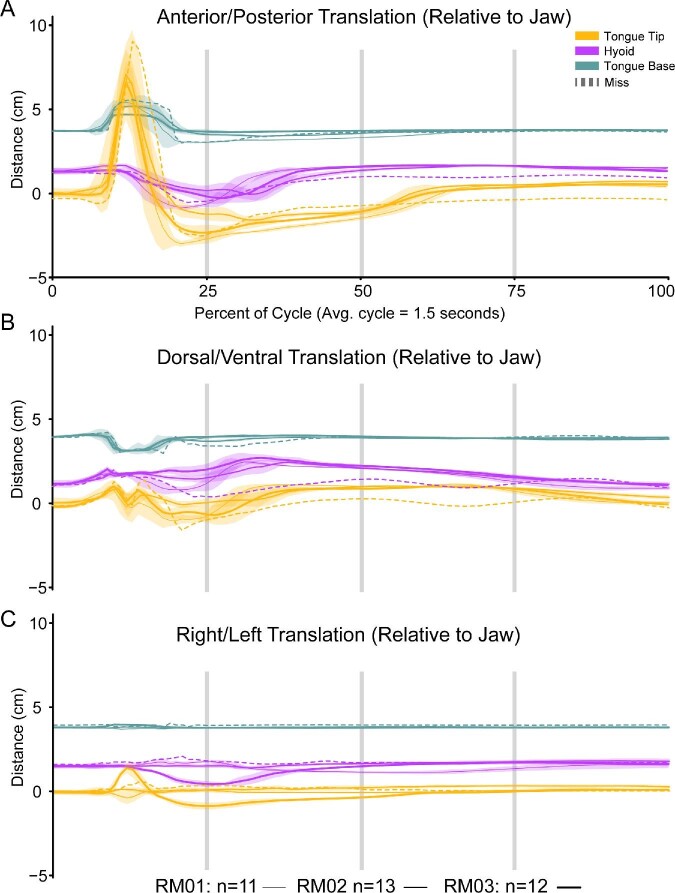
Movements of the tongue base (teal), tongue tip (yellow), and hyoid (purple) XROMM markers relative to the lower jaw along the antero-posterior axis (**A**), dorsal-ventral axis (**B**), and right-left axis (**C**). The X-axis for all plots is time as a percentage of the feeding cycle. The Y-axis is the distance that the marker is traveling relative to the jaw joint on the specified axis. The vertical gray lines indicate, 25%, 50%, and 75% through a cycle. Each colored line is the average movements from the successful feeding events for each toad (RM01 = thin line, *n* = 11; RM02 = medium line, *n* = 13; RM03 = thick line, *n* = 12). Each line is overlaid with the standard deviation of those same trials, added and subtracted from the average values at each time point. The dotted line shows the movements for a single miss event for RM01 (Event 61; Video 1).

**Fig. 6:**
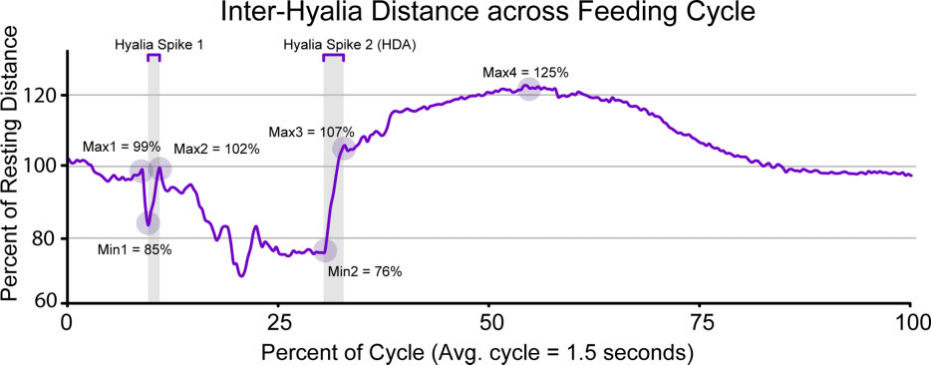
The distance between the right and left hyoid markers during a typical feeding event for RM01 (Event 17; see Video 5) as a percentage of their resting distance. The first hyalia spike event occurs around 9% through a cycle, encompassing the local minimum at this time and the local maxima to the right of it. The second hyalia spike occurs at ∼30% and includes the local minimum and the local maximum directly afterward. The last maximum occurs ∼56% through the cycle and then the hyalia return to resting distance at the end of the cycle. RM01 also experiences another spike event between the first and second events at around 19% through a cycle (coinciding with maximum tongue retraction), but this did not occur consistently in toads RM02 or RM03.

The prey capture phase encompasses the first 12.51 ± 0.15% of the feeding cycle between first head movement and maximum tongue protrusion (Table 1). During this phase, the toad closes its eyes and turns its head either to its left or right, so the prey is in line with the midline of the skull. The onset of mouth opening coincides with the onset of protrusion of the tongue tip marker (R^2^ = 0.63, *P* < 0.05; Supp. Table 6) and then is followed by slight anterior movement of the hyoid and the first local minimum distance between the right and left hyalia (Table 1; Figs. 5A and 6). The tongue rapidly reaches maximum protrusion distance, concluding the prey capture phase as the tip of the tongue contacts the prey. The tongue stretches 80.52 ± 1.97% of skull length on average at the time of maximum protrusion (Table 3).

#### Prey transport phase

**Fig. 7:**
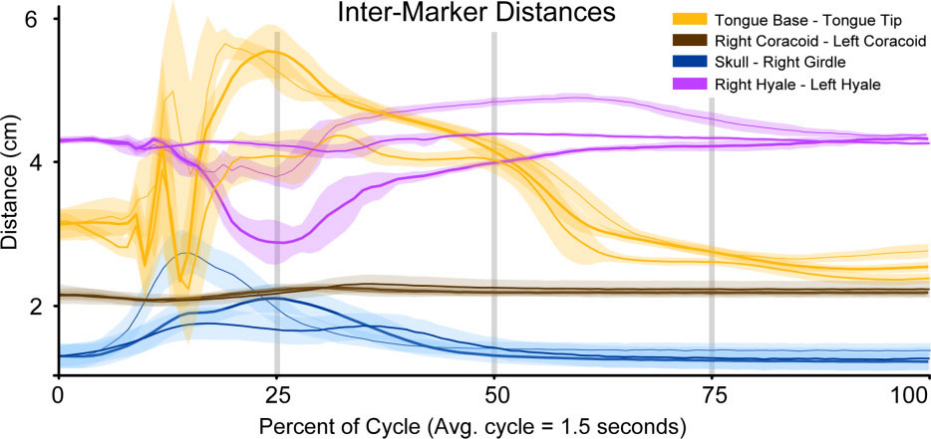
Distance changes between various pairs of markers over the course of a feeding event. The X-axis is time as a percentage of the feeding cycle. The Y-axis is the distance between the two markers of each variable in centimeters. Each colored line is the average movements from the successful feeding events for each toad (RM01 = thin line, *n* = 11; RM02 = medium line, *n* = 13; RM03 = thick line, *n* = 12). The initial position of kinematics lines along the Y-axis are arbitrary. Each line is overlaid with the standard deviation of those same trials, added and subtracted from the average values at each time point. The variables are as follows; yellow = intermarker distances between the two tongue markers, purple = intermarker distances between the two hyalia markers, blue = intermarker distances between a skull and a right girdle marker, and brown = intermarker distances between a right girdle and a left girdle marker.

**Fig. 8:**
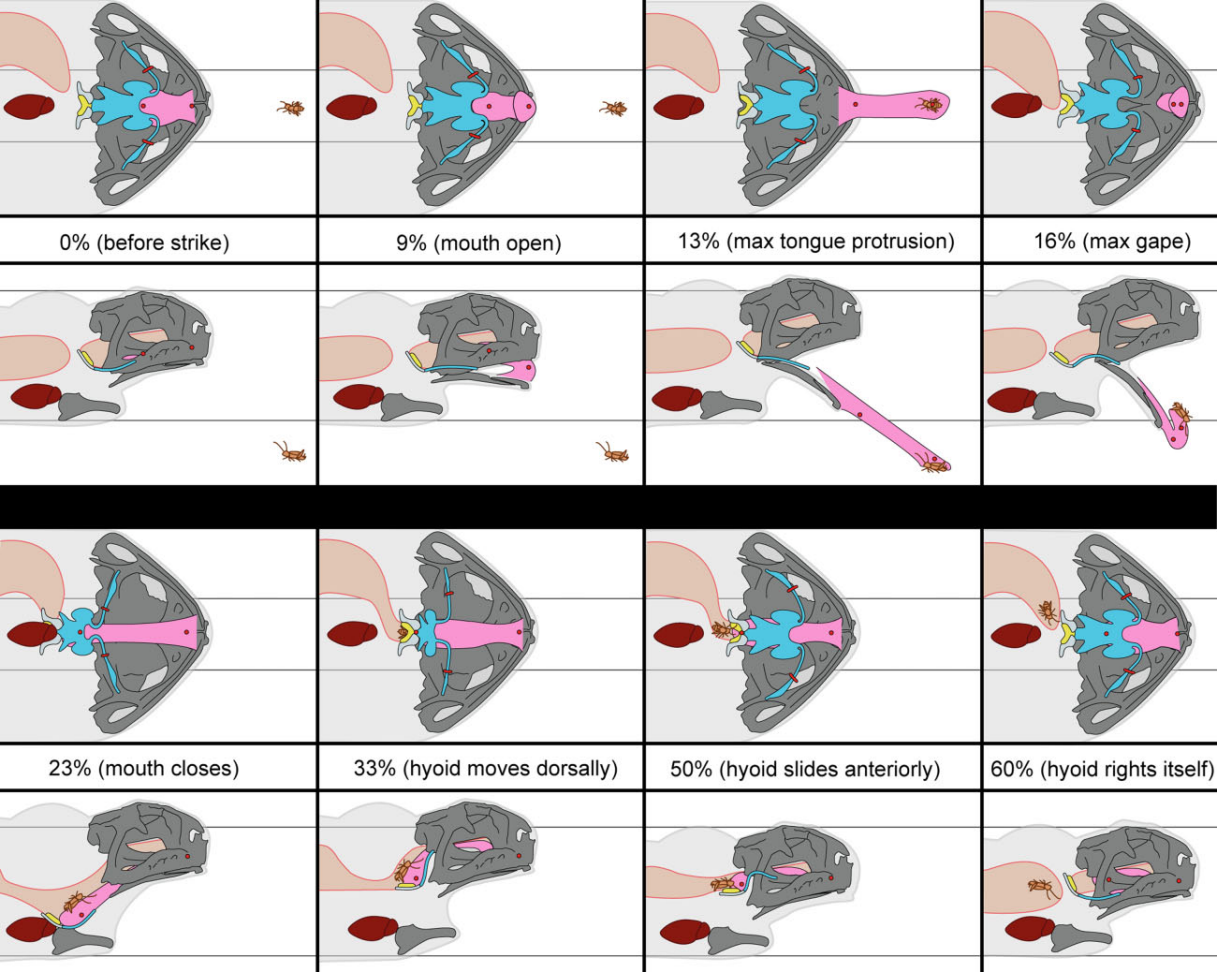
An illustration of the major events that encompass a full feeding event for *R.marina*. Upper panels are ventral views and lower panels are the same event in lateral view. Percentages listed between the upper and lower panels indicate at what % through a feeding cycle that frame occurs, followed by a brief description of the event in parentheses (see text for full description of feeding motions). The horizontal black lines of all dorsal view panels indicate the initial positions of the hyalia markers and those of all lateral view panels indicate the initial margins of the skull and pectoral girdle. Color designations are as follows: gray = bones of the skull, lower jaw, and pectoral girdle; light gray = posteromedial bones of the hyoid apparatus; blue = the cartilaginous hyoid apparatus; yellow = arytenoid cartilages of the glottis; pink = the protrusible part of the tongue; dark red = the heart; tan = the stomach and buccal cavity; red = tantalum XROMM markers; brown = the prey item. Silhouette of the toad is indicated by a gray transparency.

The tongue begins retracting immediately following maximum protrusion. Maximum gape (69.71 ± 1.17° between the axes of the skull and lower jaw; Table 3) occurs as the two tongue markers slide past each other mid-retraction (R^2^ = 0.90, *P* < 0.05; Supp. Table 6). This is followed by rapid closure of the mouth (Table 1). The distance between the skull and the pectoral girdle reaches a maximum around the same time that the mouth finishes closing (Table 1, Fig. 7). Maximum tongue retraction distance is achieved slightly before mouth closure, at which time the distal tongue tip has contacted the closed glottis posterior to the hyoid, which has been retracted to just anterior to the heart and dorsal to the pectoral girdle (Table 1; Fig. 8). The tongue stretches 87.89 ± 1.35% of skull length on average during maximum retraction (Table 3).

Toads reopen their eyes around the same time as mouth closure (Table 1). Between maximum tongue retraction and when the hyoid reaches its dorsal maximum, the hyoid apparatus rotates such that the hyoid plate is oriented parallel with the transverse plane of the toad's body (Fig. 8; Video 5; Supp. Fig. 6). As the hyoid rotates, it is pulled dorsally as the hyalia rapidly spring apart (Figs. 6 and 8; Video 5). We call this movement the “hyoid dorsal ascent” (HDA) since the hyoid moves dorsally to press the tongue closely against the roof of the mouth at its most dorsal position, against the transverse ridge formed by the pterygoid and parasphenoid bones at the back of the buccal roof. The beginning of the HDA is the second local minimum distance between the right and left hyalia and the end of the HDA is their third local maximum (Fig. 6). The hyoid markers reach the most dorsal position relative to the jaw joint at 33.86 ± 0.59% through a cycle (Table 1). The maximum yaw angle between the two halves of the pectoral girdle occurs slightly before the end of the hyoid dorsal maximum, at 29.79 ± 1.54% through the cycle.

Between 30% and 50% of the cycle, the hyoid moves anteriorly as it slides from its transverse orientation to its resting orientation (parallel with the horizontal plane of the body). The distance between the hyalia increases during this period (Fig. 6). The anterior sliding motion of the hyoid pulls both the posterior part of the hyoid and the prey up to the roof of the mouth, right at the anterior opening of the esophagus (Fig. 8). This marks the end of the prey transport phase.

#### Swallowing phase

The swallowing phase occurs directly after the prey is positioned at the pharynx, equivalent to the posterior margin of the hyoid apparatus, around 50% through a feeding cycle. Between about 50–60% through the cycle, the hyoid apparatus continues to move anteriorly, while the posterior portion of the hyoid returns to its resting position (Fig. 8), pinching the prey from the tongue tip into the front of the stomach through the muscular ring around the esophagus (see discussion for further detail of the swallowing mechanism). The swallowing phase ends after the prey has moved into the esophagus. This event generally co-occurs with the third local maximum distance between the two hyoid markers (56.48 ± 1.10% through the cycle, although this timing is variable between individuals; Table 1).

#### Recovery phase

After the prey has entered the esophagus at 56.48 ± 1.10% of the cycle, the tongue tip then slides back into its resting position in the mouth, marked by a slightly steeper incline than that during 25–50% through the cycle in its anterior-posterior kinematics plot (Fig. 5A). The hyalia slowly return to their resting length during this time (Fig. 6). The feeding cycle finishes when the tongue returns to its starting length inside the buccal cavity. We refer to the period from 56% to 100% as the recovery phase.

### Tongue elongation

We found that the tongue consistently stretches posterior to the back of the skull during the prey transport phase. The distance between the tongue base and the tongue tip markers stretches to a length of, on average, 4.14 ± 0.11 cm at maximum protrusion and 4.52 ± 0.08 cm at maximum retraction (Table 3). The mean distance that the tongue stretches during retraction equals or exceeds the mean distance that it stretches during protrusion (Table 3; Fig. 4). Maximum tongue protrusion distances spanned a large range, stretching 181.12 ± 0.07% of resting length on average, ranging from 92.17% to 264.36% across our sample (Figs. 4 and 14; Table 3; Supp. Table 2). These measures are conservative estimates of tongue stretching because the two XROMM markers were not located precisely in the distalmost tip or proximalmost base of the tongue.

Variation in maximum tongue elongation was different between retraction and protrusion. We found that retraction distances were variable across individuals (protrusion distances, *P* > 0.05; retraction distances, *P* < 0.05; Supp. Table 6), but that maximum protrusion and retraction distances were not statistically different in two individuals (RM01 and RM02, *P* > 0.05; Supp. Table 6). In one individual, the tongue was retracted further than it was protruded (RM03, *P* < 0.05; Supp. Table 6). Across all events, tongue protrusion distance is more variable than retraction distance (*P* < 0.05; Supp. Table 6). This is likely due to the variable position of the prey across trials. Tongue retraction distance shows less variation likely because the posterior movement of the tongue is constrained by hitting the closed glottis upon full retraction (see discussion section).

Retraction of the tongue occupies a large portion of each feeding cycle. On average, it takes three times longer for the tongue to fully retract after maximum protrusion (136.44 ± 7.63 ms) than for the tongue to fully protrude from rest (45.56 ± 1.84 ms). The relative timing of protrusion and retraction is similar between the tongue base and tongue tip markers, but it does take slightly longer for the tongue tip to fully retract compared to the tongue base (Table 1; Fig. 5A). The tongue base marker moves out of the mouth with the tongue tip marker but spends longer at the maximum protrusion distance than does the tongue tip marker (Fig. 5A). In one of our toads (RM01), the tongue base marker also experiences a second, much smaller local maximum at around 30% through the feeding cycle which coincides with the hyoid dorsal maximum (Table 1; Fig. 3). The HDA likely stretches the tongue slightly as it presses the distal tip of the tongue against the roof of the mouth.

The maximum tongue stretch distance during protrusion varies between 2.7 cm and 5.3 cm (see Supp. Table 2). In general, the longer the tongue stretches to catch the prey, the longer the prey capture phase takes of a feeding cycle (R^2^ = 0.28, *P* < 0.05; Supp. Table 6).

### Hyoid movement

The hyoid apparatus moves considerably during the feeding cycle, with most of its major movements occurring during the prey transport and swallowing phases (Figs. 5–8). The hyoid apparatus is cartilaginous and flexible; it does not behave like a rigid structure. For this reason, we present both general translations of the hyoid markers relative to the lower jaw (Fig. 5) as well as changes in the distance between the right and left hyoid markers over the course of a feeding cycle (Fig. 6).

Throughout a typical feeding cycle, the distance between the two hyoid markers ranges from 40–132% of their resting distance (2.63 ± 0.17 cm; Fig. 6; Supp. Table 2). We recovered two consistent events of rapid distance change, which we will refer to as a “hyalia spikes.” The onset of the first spike (Hyalia Min 1; see Table 1, Fig. 6) coincides with the onset of tongue protrusion and the second begins at 29% through a cycle, slightly after the moment of maximum tongue retraction (Hyalia Min 2; see Table 1, Fig. 6). The first hyalia spike is brief (duration: 18.33 ± 5.9 ms). The second hyalia spike covers more distance and is also slower (duration: 89.22 ± 5.9 ms). This second spike event coincides with the HDA and the hyalia reach their dorsal maximum relative to the lower jaw (Fig. 5B) at this time (see Table 1). In one individual (RM01), we observed another small spike event between these two events (at ∼19% of the cycle). After the HDA, the distance between the two hyoid markers continues to steadily increase until it reaches a maximum distance (2.88 ± 0.1 cm, on average) at ∼56% through the cycle (although not for RM03, see below) and then steadily decreases to the resting distance as the feeding cycle finishes.

**Fig. 9:**
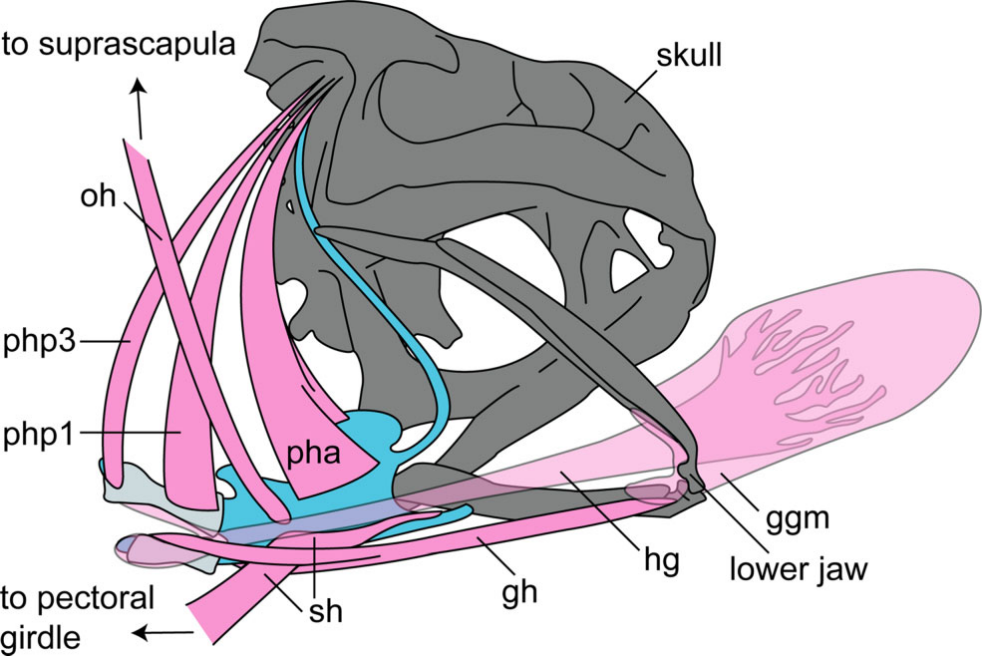
An illustration of select hyoid musculature in posteroventral view for *R.marina* with the tongue extended. Hyoid cartilages are blue, posteromedial bones light gray, skull and jaw bones are dark gray, muscles pink, tongue muscles semitransparent pink. For simplicity, petrohyoidues and omohyoideus muscles illustrated for only the right side, sternohyoideus and geniohyoideus muscles illustrated only for the left side. Abbreviations: gh = m. geniohyoideus; ggm = m. genioglossus medials; hg = m. hyoglossus; oh = m. omohyoideus; pha = m. petrohyoideus anterior; php1 = m. petrohyoideus posterior primus; php3 = m. petrohyoideus tertius; sh = m. sternohyoideus. Muscle insertions referenced from Ecker 1889; Trewavas 1932.

In addition to the periods of rapid distance change between the hyalia, the hyoid markers also translate relative to the lower jaw during a feeding cycle (Fig. 5). During the prey capture phase, the hyoid markers move slightly anterodorsally on average (0–12% of the cycle, Fig. 5). Because the hyalia are flexible and attached to the otic region of the skull (Fig. 9), anterior translation of the hyoid plate pulls the two hyoid markers medially, decreasing the distance between them to produce the first hyalia spike event (Fig. 6). At this same moment, the angle between the hyoid plate and the posteromedial bones increases (Video 5; Supp. Fig. 6, frame 50).

Most of the hyoid movements occur after the end of the prey capture phase. Between the time of maximum tongue protrusion and maximum gape, the hyoid plate moves posteroventrally towards the pectoral girdle (Video 5; Supp. Fig. 6, frame 61), posterior relative to the lower jaw (Fig. 5A). The hyoid continues to move posteroventrally until its posterior portion (the posteromedial bones, as well as arytenoid cartilages and associated musculature) abuts the heart (Video 5; Supp. Fig. 6, frame 78; Fig. 8, 23%). At this point, the angle between the hyoid plate and the posterior portion decreases, forming an “L” shape in lateral view. Also at this moment, the flexible hyalia (and XROMM markers) now point medially to accommodate the posteroventral movement of the hyoid plate. This medial rotation causes the distance between the markers to decrease, which marks the beginning of the second hyalia spike. Following this, the “L” shape of the hyoid rotates counterclockwise about 30° in lateral view as the HDA occurs (Video 5; Supp. Fig. 6, frame 105), with the hyoid markers rapidly moving apart as they move dorsally and anteriorly in the mouth, antero-dorsally relative to the lower jaw (Fig. 5). At the end of the HDA, the hyoid markers reach their maximum dorsal position relative to the jaw joint (Fig. 5B) and approach close to the skull (Video 5; Supp. Fig. 6, frame 105). The tongue is squeezed tightly between the hyoid and the skull as this happens.

Following the HDA, the posterior part of the hyoid continues to move dorsally as the anterior part of the hyoid moves anteriorly as the distance between the hyalia markers increases (Video 5; Supp. Fig. 6, frame 122; Figs. 5 and 6). The posterior part of the hyoid presses against the roof of the mouth, just ventral to the tip of the tongue. Roughly simultaneous with the final maximum distance between the hyalia (56.48 ± 1.10% through a cycle; Table 1), the posterior part of the hyoid apparatus rotates slightly clockwise in lateral view (Video 5; Supp. Fig. 6, frame 187) which squeezes the prey off of the tip of the tongue. The prey then moves further into the alimentary canal (Video 5; Supp. Fig. 6, frame 250) and the tongue tip and hyoid markers slide antero-ventrally (Fig. 5) to their resting positions. It takes the remaining 40% of the cycle duration for the tongue and the hyoid markers to return to their resting positions.

### Pectoral movement

**Fig. 10:**
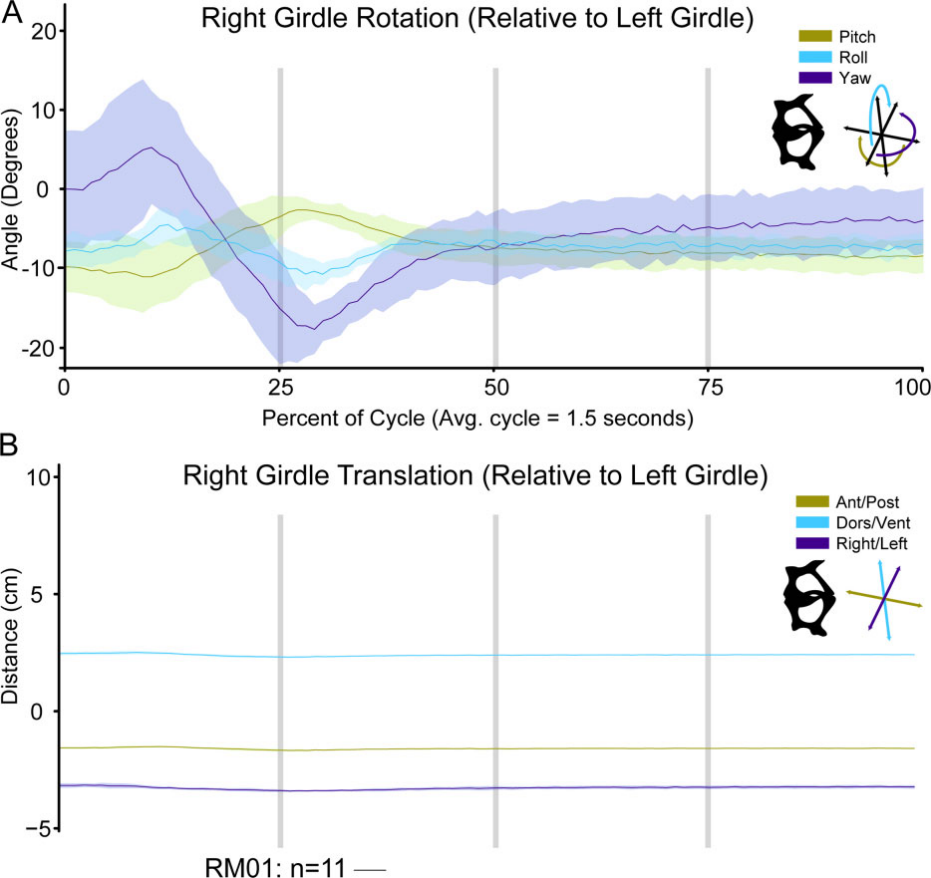
Movements of the right half of the pectoral girdle relative to the left half throughout a feeding cycle. (A) Rotations depicted above and (B) translations below. The X-axis for both plots is time as a percentage of the feeding cycle. In A, the Y-axis is the angle in degrees that the right girdle is rotating relative to the left girdle. In B, the Y-axis is the distance in centimeters that the right girdle is translating relative to the left girdle. The initial position of kinematics lines along the Y-axis are arbitrary. The vertical gray lines indicate, 25%, 50%, and 75% through a cycle. Each colored line is the average movements from the successful feeding events for RM01 (*n* = 11). Each line is overlaid with the standard deviation of those same trials, added and subtracted from the average values at each time point. Antero-posterior and pitch movements are indicated in lime green, dorsal-ventral and roll movements are indicated in light blue, and right-left and yaw movements are indicated in dark purple. A silhouette of the right and left halves of the pectoral girdle and orientation schematic are illustrated under the legend.

We found evidence that the two halves of the pectoral girdle translate slightly relative to each other throughout the feeding cycle (Fig. 10B; Supp. Table 2), although the translations between girdle halves were close to their precision thresholds. The distance between the two XROMM markers embedded in the distal right and left coracoid bones decreases slightly (0.2 cm) during tongue protrusion and then stretches back to near resting distance following mouth closure (Fig. 7). Additionally, our observations of the Maya animations for RM01 show that the pectoral girdle translates anteriorly to its starting position as the frog leans forward to catch the prey. The girdle moves slightly anteriorly during prey capture and sometimes greatly anteriorly when a lunging behavior is performed (Supp. Fig. 7).

The right and left halves of the pectoral girdle also rotate slightly relative to each other during a feeding event (Fig. 10A). Of our three individuals, only one (RM01) had pectoral girdle movements relatively unaffected by tracking noise from X-ray video so we plotted only that individual in Fig. 10. We found rotations of around 30° of yaw (e.g., anterior-most tips of the clavicles rotate together, towards the midline) occur from the beginning of the cycle until around 50% of the cycle (Fig. 10A). The two halves of the girdle experience small (around 5°) rotations of pitch and roll during this same period. Average maximum yaw for RM01 was 19.74° and occurred at around 29% through a cycle.

### Skull and jaw movement

**Fig. 11:**
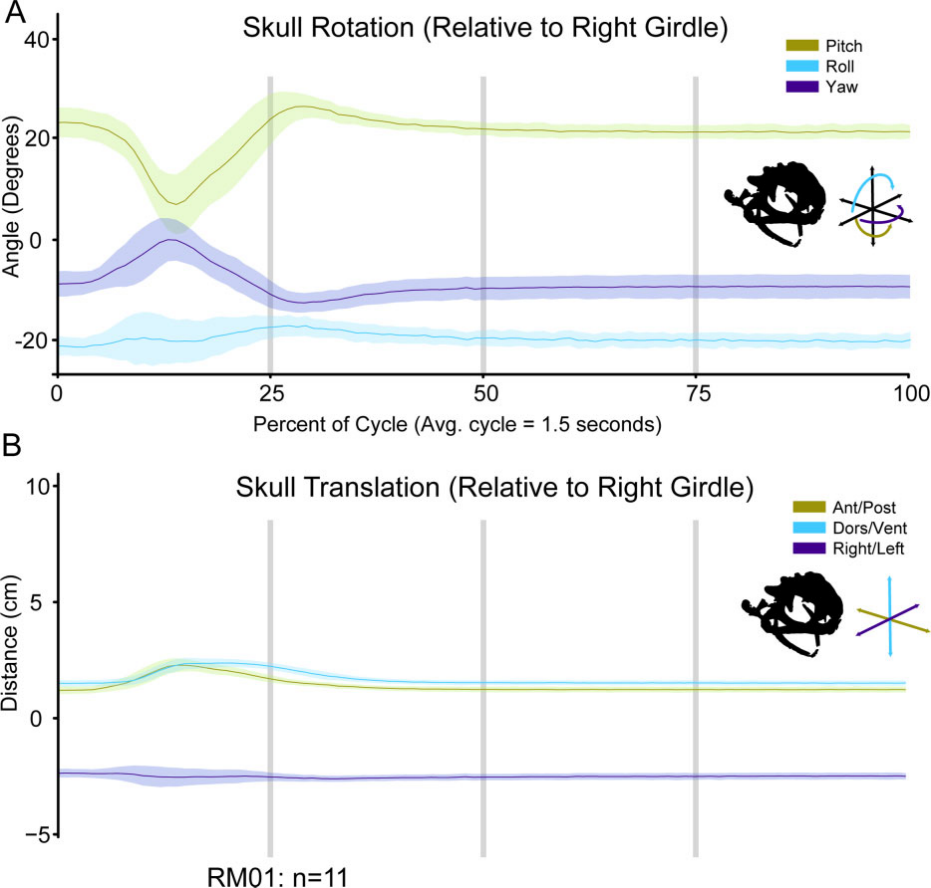
Movements of the skull relative to the right half of the pectoral girdle throughout a feeding cycle. (A) Rotations depicted above and (B) translations below. The X-axis for both plots is time as a percentage of the feeding cycle. In A, the Y-axis is the angle in degrees that the skull is rotating relative to the right girdle. In B, the Y-axis is the distance in centimeters that the skull is translating relative to the right girdle. The initial position of kinematics lines along the Y-axis are arbitrary. The vertical gray lines indicate, 25%, 50%, and 75% through a cycle. Each colored line is the average movements from the successful feeding events for RM01 (*n* = 11). Each line is overlaid with the standard deviation of those same trials, added and subtracted from the average values at each time point. Antero-posterior and pitch movements are indicated in lime green, dorsal-ventral and roll movements are indicated in light blue, and right-left and yaw movements are indicated in dark purple. A silhouette of the skull, lower jaw, and orientation schematic are illustrated under the legend.

Relative to the pectoral girdle, the skull both rotates and translates during a feeding cycle (Figs. 7 and 11). Most of these movements occur in the first half of a cycle, with only slight motions occurring after the HDA. On average, the distance between the skull and pectoral girdle increases to 0.93 ± 0.07 cm at 23.32 ± 1.38% through a cycle, right after maximum tongue retraction has occurred (Tables 1 and 3). Prior to this point, the skull pitches upward and moves anterodorsally relative to the pectoral girdle as the tongue is thrown out of the mouth and then retracted (Video 5; Fig. 11). Following the maximum stretching between skull and girdle, the skull pitches back downward as the HDA occurs and the skull translates posteroventrally, back to resting position (Video 5; Fig. 11).

**Fig. 12:**
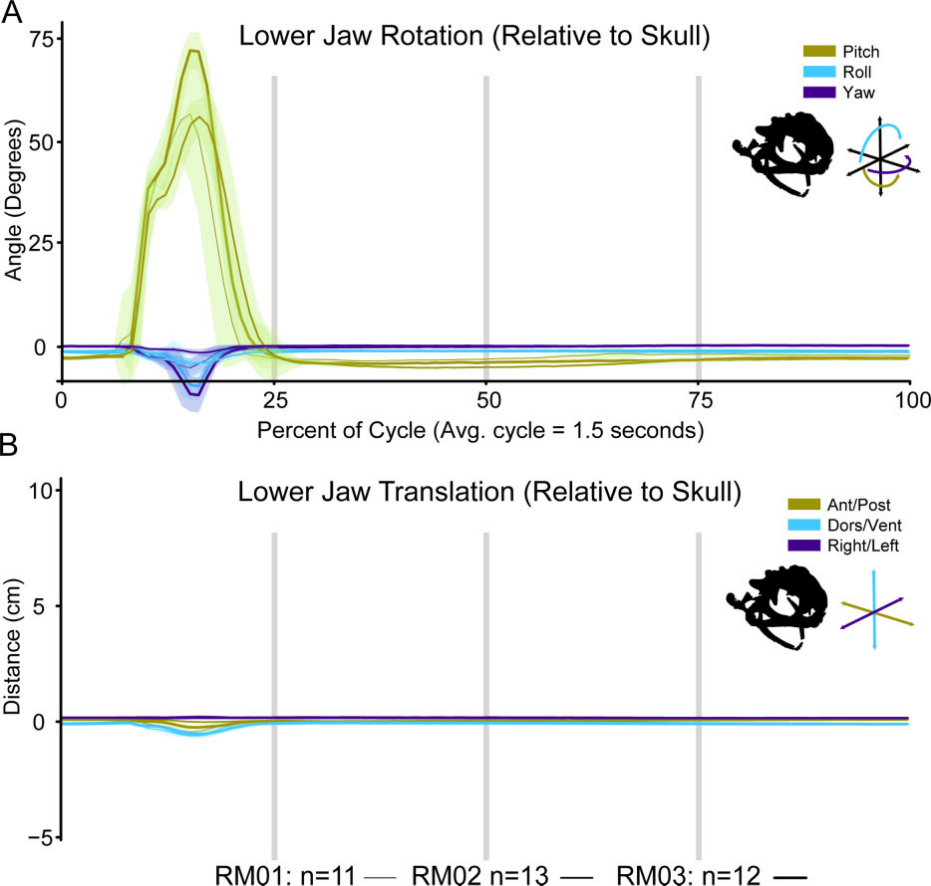
Movements of the lower jaw relative to the skull throughout a feeding cycle. (A) Rotations depicted above and (B) translations below. The X-axis for both plots is time as a percentage of the feeding cycle. In A, the Y-axis is the angle in degrees that the jaw is rotating relative to the skull joint. In B, the Y-axis is the distance in centimeters that the jaw is translating relative to the skull joint. The initial position of kinematics lines along the Y-axis are arbitrary. The vertical gray lines indicate, 25%, 50%, and 75% through a cycle. Each colored line is the average movements from the successful feeding events for each toad (RM01 = thin line, *n* = 11; RM02 = medium line, *n* = 13; RM03 = thick line, *n* = 12). Each line is overlaid with the standard deviation of those same trials, added and subtracted from the average values at each time point. Antero-posterior and pitch movements are indicated in lime green, dorsal-ventral and roll movements are indicated in light blue, and right-left and yaw movements are indicated in dark purple. A silhouette of the skull, lower jaw, and orientation schematic are illustrated under the legend.

The lower jaw both rotates and translates relative to the skull. Nearly all motions of the jaw relative to the skull occur prior to 25% through a feeding cycle (Fig. 12). As the mouth opens, the jaw translates slightly posteroventrally relative to the skull, and then anterodorsally as it closes (Fig. 12B). In all three individuals, the jaw pitches downward rapidly as the tongue extends out of the mouth, pauses at this angle (∼40°) briefly before rapidly pitching downward even further to maximum gape (69.71 ± 7°) as the tongue slides back into the mouth (Fig. 12A). Immediately after maximum gape, the jaw pitches upward equally rapidly until the mouth is fully closed at around 23.63 ± 2.2% through a feeding cycle. In one event (RM03, event 07_1, a double-swallow) the mouth briefly opens to about 5° of pitch when the tongue is readjusted in the front of the buccal cavity, but generally the mouth stays closed for the remainder of the feeding cycle.

### Individual variation, missed strikes, and double swallows

**Fig. 13:**
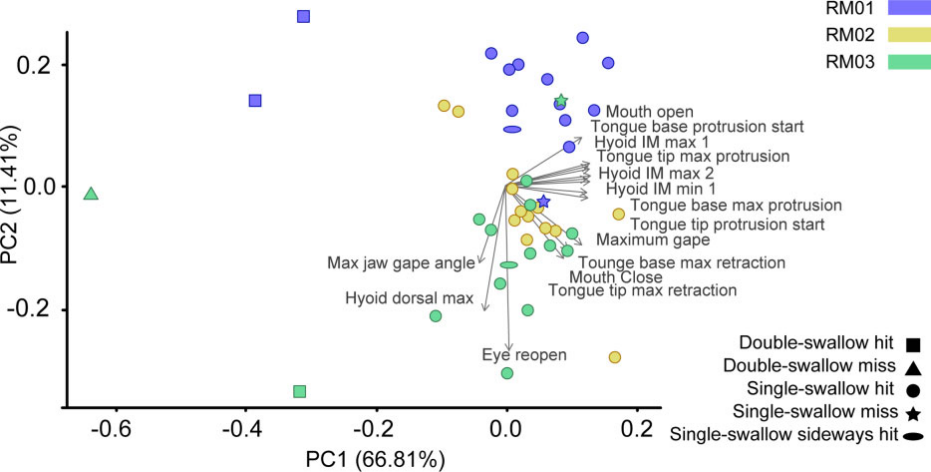
A plot of the first two principal components of a PCA showing the variation in major kinematic timing variables within our feeding event dataset (*n* = 44). RM01 events are plotted in purple, RM02 in yellow, and RM03 in green. Single-swallow, straight, successful events are shown as circular points, double-swallow, straight, successful events are shown as squares, double-swallow, straight, unsuccessful events are shown as triangles, single-swallow, straight, unsuccessful events are shown as stars, and single-swallow, sideways, successful events are shown as ovals. Eigenvectors are marked and labeled from the origin point in gray. Variables are all timing values calculated as cycle percentages except for max jaw gape which is in degrees.

Our PCA of major kinematic variables shows that most feeding events are similar, with double-swallow events more distinct from typical events (single-swallow hits) than misses or sideways strikes (Fig. 13). The first principal component (PC1) accounted for 66.81% of total variance and the second principal component (PC2) accounted for 11.41% of the variance. Most of the variance appears to be a result of the extended timing of double-swallow and delayed-swallow events, four of which are far removed from the other, single-swallow events on PC1. The results of the PCA also show that RM01 is somewhat distinct from RM02 and RM03 on PC2, although this axis encompasses less variation.

We corroborated the results of the PCA with a series of correlation matrices among these same variables, with detailed comparisons in the following sections.

#### Variation between toads

**Fig. 14:**
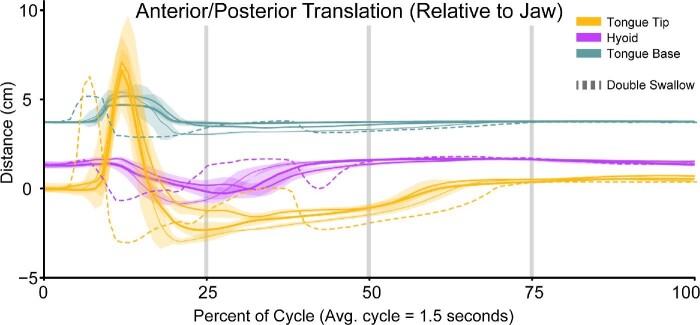
Movements of the tongue base (teal), tongue tip (yellow), and hyoid (purple) XROMM markers relative to the lower jaw along the antero-posterior axis. The X-axis is time as a percentage of the feeding cycle. The Y-axis is the distance that the marker is traveling relative to the jaw joint in the specified axis. Each colored line is the average movements from the successful feeding events for each toad (RM01 = thin line, *n* = 11; RM02 = medium line, *n* = 13; RM03 = thick line, *n* = 12). Each line is overlaid with the standard deviation of those same trials, added and subtracted from the average values at each time point. The dotted line shows the movements for a single double-swallow event for RM01 (Event 8; Video 3).

Overall, we find that movements of the tongue, hyoid, skull, jaw, and pectoral girdle are highly cross-correlated during typical (successful, single-swallow, straight strikes) feeding cycles for our three individuals (Table 4). Of 12 measures of average translations between these structures, excluding right and left translations, 10 were highly correlated (Pearson's Correlation coefficient > 0.7) between all three individuals (Table 4; see Figs. 5, 12, 14). Right and left translations are not highly correlated, but this is expected since most feeding motions are aligned with the midline of the body. Regarding rotational movements, jaw rotations relative to the skull are highly correlated on all axes and skull rotations relative to the girdle are highly correlated in only one axis (pitch). Rotations between the two halves of the pectoral girdle are not highly correlated (Table 4). Changes in intermarker distances between the two tongue markers, skull and girdle markers, hyoid markers, and right and left distal coracoid markers (Fig. 7) were all highly correlated except for the hyoid markers which were only moderately correlated (Pearson's correlation coefficient = 0.53). Some variation between individuals may be due to the XROMM markers not being in the exact same location in each toad. Some variation may also have been introduced as a result of slight size difference between the three individuals (RM01 = 23.8 cm SVL; RM03 = 20.32 cm SVL; RM02 = 19.59 cm SVL).

#### Variation between misses and hits

From our full dataset of recorded videos, we determined that the toads successfully caught the prey in 93.5% of attempts (93.62% for RM01, *n* = 93; 93.58% for RM03, *n* = 109).

Our dataset included two no-swallow misses (Video 1) and one delayed-swallow miss (Fig. 5; Supp. Table 2). In general, a no-swallow miss is similar kinematically to a hit except that an HDA motion does not occur after tongue retraction (see Fig. 5). For the same 12 translation variables described above, comparing misses to average hits from the same toad, eight were highly correlated (Table 4). The only two variables that differed in a miss were dorsal-ventral movements of the hyoid and tongue tip markers. This difference is likely a consequence of skipping the HDA motion. Patterns of correlation in the rotation variables are similar to those that we observed in individual variation. Measures of intermarker distance changes were all highly correlated (Table 4).

In contrast, none of the kinematic variables of the delayed-swallow miss correlate strongly to those of a typical hit (Table 4; Supp. Table 5), with the highest correlation coefficient being 0.47 for dorsal-ventral translations of the hyoid relative to the jaw. Overall, this event correlates the least to typical events out of all the atypical events we examined. This is expected since this event differs from a typical event in two different ways (being both a miss and a delayed swallow).

#### Variation in double and delayed swallows

Our dataset also included two double-swallow successful events (Video 3) and one delayed-swallow successful event (Video 4). A double or delayed swallow differs from a typical strike in that the toad performs an additional, delayed movement of the hyoid apparatus well after closure of the mouth (Fig. 14). This extra movement extends the length of the cycle significantly (*P* = 2e-16; Supp. Table 6) and warps the proportions of events in a full cycle (Fig. 14). In our cross-correlation matrices, a double-swallow has overall low correlation values when compared pairwise to typical events (Table 4). Only three variables out of 27 translation and rotation measures were highly correlated. All four measures of intermarker distance changes were highly correlated.

The delayed-swallow successful event differs from the double-swallows in that the toads readjust the tongue in the front of the mouth before swallowing rather than simply performing two HDAs (compare Videos 3 and 4). For the delayed-swallow hit, again, only three variables out of 27 translation and rotation measures were highly correlated (two of which were different than those correlated in the double-swallows). The variables measuring intermarker distance change were only slightly less cross-correlated than those of the double-swallows.

#### Variation between side and straight strikes

In two cases, the toads moved the skull 30° to the side of the midline to perform a side strike (Video 2). Kinematically, side strikes are highly correlated with typical strikes from the same individual (Table 4). Of the 12 measures of translation motions, 11 were highly correlated with average, straight strikes from the same toad (Table 4). Of the nine rotation variables, three were highly correlated with typical strikes from the same toad. Three of the four measures of intermarker distance changes were highly correlated.

The swinging movement of the head does not appear to cause the tongue to move significantly differently compared to the movements of a straight strike. Overall, side strikes differ less from typical strikes than the other atypical conditions we examined (double-swallows, delayed-swallows, and misses).

### Dissection results

At rest, the hyoid sits ventrally in the buccal cavity where the distal ends of the hyalia are attached to the otic region of the skull (Figs. 8 and 9). The back of the hyoid apparatus supports the two posteromedial bones, between which sit the folds of the glottis and the muscles of the larynx. Directly dorsal to the glottis is a short esophageal region that opens to the stomach (Fig. 8). The walls of the buccal cavity and esophagus are elastic, and there is a shallow, ridged texture of the buccal wall oriented parallel to the sagittal plane in the buccal cavity near the pharynx. The hyoid itself is thin (0.5 mm) and pliable like a sheet of laminate paper.

The back of the throat can be moved at least half a head length anteriorly and posteriorly from its resting position (Supp. Table 1). The back of the throat typically rests just posterior to the skull but can be physically pushed back by clasping the front of the hyoid plate with tongs and pushing the hyoid apparatus posteriorly. Doing this pushes the glottis region back against the anterior part of the heart, towards the posterior margin of the coracoids and well behind the back of the skull. In this conformation, the hyalia are pulled taut, the anterior-most loops of the hyalia rotate to point together medially (as in Fig. 8; 23%), and the distal edges of the hyalia rotate from anteriorly-pointed to posteriorly-pointed. The hyoid plate normally sits flat when at rest but can be manipulated to be curled dorsally or ventrally easily by gripping it on its anterior and posterior margins with tongs and pushing the margins together.

Additionally, we found that the pterygoid and parasphenoid bones form a transverse ridge across the roof of the mouth which protrudes into the buccal cavity, in the same area that the hyoid markers nearly touch in the Maya animations. When the eyes are manually closed and pushed ventrally into the skull, they descend shallowly into the mouth cavity. However, the eyes are anterior to this lateral bar and well anterior to the hyoid when it is in its posteriorly pushed conformation. For this reason, it is unlikely that they are used to push (small) prey into the throat during swallowing (see discussion).

## Discussion

This work is the first to provide detailed descriptions of prey transport, swallowing, and recovery movements during the feeding cycle of *R. marina*. Our findings of the motions of prey capture corroborate previous studies (Gans and Gorniak 1982a, 1982b; Trueb and Gans 1983; Matsushima et al. 1985; Deban and Nishikawa 1992; Nishikawa and Gans 1996; Nishikawa et al. 1999; Wolff et al. 2004; Lappin et al. 2006; Monroy and Nishikawa 2009) but demonstrate that the feeding cycle is longer (1.52 ± 0.28 s on average) and more complex than previously reported. Our findings illuminate in detail the functional implications of hyoid movement during prey manipulation and reveal how the tongue stretches during prey transport. The hyoid apparatus performs a scraping motion against the roof of the mouth following mouth closure, which positions the prey in the pharynx. The hyoid is then pulled anteriorly and returns to its resting position, which pushes the prey off the tongue and into the esophagus. The tongue was found to consistently stretch posterior to the back of the skull during prey transport. The tongue then slides forward and takes about 670 ms to recover from stretching posteriorly (Fig. 3; Supp. Table 2; Video 5). In the following sections, we will describe the functional and evolutionary implications of these new findings in further detail.

### Hyoid movement and function

The movements and functions of the hyoid apparatus during feeding behaviors in *R. marina* have historically been a topic of debate (see Emerson 1977; Gans and Gorniak 1982a, 1982b; Nishikawa 2000), but current understanding posits that the hyoid does not participate in the tongue protrusion mechanism and few papers discuss its function in the later phases of the feeding cycle. We provide evidence that the hyoid apparatus moves dynamically during prey transport and swallowing behaviors, which may indicate a previously unexplored functional role during feeding behaviors of cane toads and potentially more broadly across the diversity of frogs. The apparent requirement of the HDA motion (where the hyoid rapidly translates dorsally) for successful swallows suggests that the hyoid apparatus plays an important role in positioning prey at the anterior margin of the pharynx prior to swallowing (Fig. 8). Equally consistent, the anterior sliding and righting of the hyoid following the HDA appears to aid in prey removal from the sticky tongue pad via squeezing the tongue against the roof of the mouth. The dynamic motions of the hyoid during prey transport and swallowing indicate that it plays an important role during the feeding cycle and offer recontextualization of known buccal anatomy. We acknowledge, however, that our interpretations of hyoid movements are hypotheses based on a combination of measurement methods which do not involve direct observation of this complex structure. More concrete support from EMG data of hyoid musculature is an important next step to understand movement of the hyoid.

**Fig. 15:**
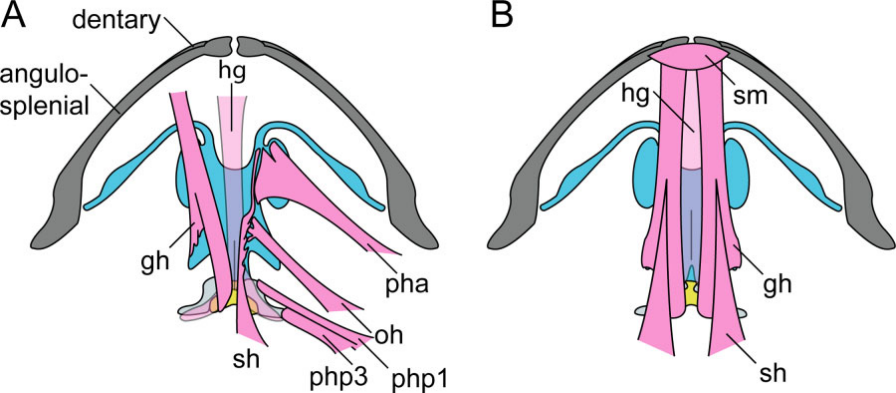
An illustration of select hyoid musculature in ventral view for *R.marina*, with (A) showing insertion areas and (B) showing the *in vivo* positions of the muscles. Hyoid cartilages are blue, posteromedial bones light gray, arytenoid cartilages yellow, jaw bones are dark gray, muscles pink. Abbreviations: gh = m. geniohyoideus; hg = m. hyoglossus; oh = m. omohyoideus; pha = m. petrohyoideus anterior; php1 = m. petrohyoideus posterior primus; php3 = m. petrohyoideus tertius; sh = m. sternohyoideus; sm = m. submentalis. Muscle insertions referenced from dissections and Ecker 1889; Trewavas 1932.

The hyoid apparatus is nested within a dynamic system of musculature that provides fine control of the prey in the buccal cavity. The only points of articulation between the hyoid and a rigid structure are the attachments of the distal ends of the hyalia to the prootic cartilages of the skull (Ecker 1889). The hyoid is suspended in the buccal cavity like a marionette on strings, precisely animated by the many muscles holding its position (Figs. 9 and 15). The petrohyoideus muscles are believed to constrict the pharynx (Székely and Matesz 1993), but also may provide control over dorsal movements of the hyoid plate via sequential activation. Another muscle that may pull the hyoid dorsally is the mm. omohyoideus, which arises from the ventral scapula and inserts on the hyoid plate. The mm. intermandibularus joins the two halves of the lower jaw; a posterior portion near the jaw joint arises from the cartilaginous hyalia and is often referred to as the m. interhyoideus (Duellman and Trueb 1986). The m. interhyoideus is thought to play a role in swallowing (mm. submaxillaris of Ecker 1889). In our X-ray videos, we observed that the posterior part of the buccal floor moves posterodorsally during the HDA (Video 5), which might result from contraction of the m. interhyoideus that forces the hyalia to ascend more dorsally than they would otherwise. The mm. geniohyoideus likely pulls the hyoid anteriorly during this phase because it arises on the anterior part of the lower jaw and inserts on the ventral hyoid plate (Duellman and Trueb 1986). Previous EMG data showed that the mm. sternohyoideus, hyoglossus, geniohyoideus, and submentalis are active following tongue retraction, but it is not known how this activity coincides with the HDA (Gans and Gorniak 1982a, 1982b). Further experimental studies are needed to determine the function of these muscles during prey manipulation and swallowing.

Additionally, we found evidence that there may also be an elastic component of hyoid movement during prey transport. Through our dissections, we found that the hyoid cartilages are pliable and return to the resting conformation after being manually distorted. The rapid motions of the hyalia during the two major spike events (Fig. 6) may be elastically mediated during the feeding cycle. Hyalia spike 1 appears to be caused by the hyalia bouncing ventrally against the floor of the mouth as the jaw rapidly opens, which briefly pulls the hyalia closer together before they spring apart (Video 5; Fig. 8). Hyalia spike 2 occurs after the hyoid has been pulled posteroventrally, which pulls the hyalia close together out of their resting conformation (Fig. 8). Emerson (1977) postulated an elastic component to hyoid movements during prey capture, and while the specifics of that model were later disproven (Gans and Gorniak 1982b), elastic recoil of the hyoid might still be an important component of prey manipulation and swallowing. Further evidence such as muscle activation data is needed to more concretely understand any elastic components of the hyoid during feeding. While movement of the hyoid seems to be critical for prey manipulation and swallowing, Gans and Gorniak (1982b) reported that *R. marina* feeds normally when the hyoid is immobilized. They did not specify whether their wiring scheme prevented the hyoid from moving posteriorly or dorsally, only that it prevented hyoid protraction. However, because these conclusions were based on analysis of light videos, movements after the mouth closed would not have been possible to observe, so potential impacts on prey manipulation and swallowing might still have occurred even if other movements appeared normal.

### Tongue movement and function

Our findings show for the first time the extensive elongation of the tongue not only during the prey capture phase of feeding by *R. marina*, but also during the prey transport phase (Fig. 4). Overall, our results corroborate the findings of previous studies regarding tongue movements and functions during protrusion (Emerson 1977; Gans and Gorniak 1982a, 1982b; Nishikawa and Gans 1996) but we found many novelties following the completion of protrusion. We observed that the tongue stretches posterior to the back of the skull during the prey transport phase (Fig. 8; Video 5). The tongue stretches, on average, more during maximum retraction than it does during maximum protrusion (Fig. 4), and its retraction distance is less variable than its protraction distance. The extreme retraction of the tongue appears to position the prey in an optimal location for further intrabuccal-manipulation and swallowing (see section below). In a typical events where the prey does not reach this position, the toad rearranges the tongue and prey within the mouth until the prey and tongue are positioned at this maximally retracted position (Supp. Fig. 3, Video 3). The fact that the toad maximally retracts its tongue even in the absence of tongue protrusion suggests that this extreme retraction of the tongue is mechanically important for toad feeding behaviors.

Mechanically, tongue retraction seems to be facilitated by the dynamic, posteroventral translation of the hyoid apparatus (Fig. 8; Video 5). Our current understanding of tongue retraction mechanics is that the m. hyoglossus functions to pull the tongue back into the mouth, perpendicular to the interface between the tongue and the prey (Gans and Gorniak 1982a; Ritter and Nishikawa 1995; Tso et al. 1995; Nishikawa 2000; Kleinteich and Gorb 2014). Our data does not contradict this idea, but the posteroventral movement of the hyoid that occurs during retraction suggests that while the m. hyoglossus pulls the tongue toward the hyoid, the mm. sternohyoideus is pulling the hyoid toward the pectoral girdle (see Figs. 8 and 9). Both muscles are likely important in achieving maximum tongue retraction, as without the posteroventral movement of the hyoid, the tongue would not travel as far posteriorly. Indeed, the position of the hyoid apparatus appears to impede further retraction of the tongue during this phase of the feeding cycle. The tongue tip and its attached prey slap into the ventral curve of the hyoid apparatus, which supports the closed glottis, much like a ball hitting a glove (see Fig. 8). This anatomical constraint is likely what limits variation in the distance that the tongue stretches during maximum retraction (Figs. 4 and 8).

One question that is underscored by our results is, what role does the tongue play in prey manipulation and prey removal mechanisms? Previous work hypothesized that the tongue is the primary element involved in prey transport within the buccal cavity (Regal and Gans 1976; Gray et al. 1997; Nishikawa 2000) but lacked experimental evidence for this. In contrast, our results show that the tongue's movements within the buccal cavity are tightly coordinated with those of the hyoid plate (Fig. 5; Videos 1‒5). In events that include prey position readjustments, the hyoid is involved in moving the tongue anteriorly and posteriorly in the mouth even in isolation from tongue protrusion motions (Supp. Fig. 3; Video 3). Additionally, the hyoid plate always presses the tongue against the roof of the mouth during the swallowing phase (Fig. 8). This appears necessary for prey removal from the tongue and appears to hold the tongue in a stretched position for longer than it typically would be without the HDA motion (compare a hit and a miss, Videos 1 and 5). Potentially, the tongue may actively shorten to aid in its detachment from the prey, but activation of the tongue muscles may not necessarily be required to achieve prey removal. The swallowing and recovery phases involve the tongue shortening at a steady, relatively slow rate following the HDA (Figs. 5 and 7), which may indicate that it is moving passively via its own elastic recoil. Further EMG work examining these phases of the feeding cycle will be necessary to confirm whether the tongue plays an active or passive role in intrabuccal manipulation, prey removal, and recovery mechanisms.

### Intrabuccal prey manipulation and swallowing

The results of this work provide novel insights into the mechanisms of prey manipulation and swallowing in cane toads. As mentioned, most prior studies on *R. marina* tongue mechanics focused on lingual protrusion (Emerson 1977; Gans and Gorniak 1982a, 1982b; Nishikawa and Gans 1996) and there are limited data about events following mouth closure. Some studies hypothesized that the tongue plays a dominant role in intrabuccal prey manipulation (Regal and Gans 1976; Gray et al. 1997; Nishikawa 2000), but we find that the hyoid apparatus, tongue, and associated musculature all coordinate to position the prey at the front of the pharynx prior to swallowing. Our results show consistent motions of the hyoid apparatus occur prior to swallowing (HDA), and that precise positioning of the tongue tip and prey are needed to initiate a swallow. In events where the prey is out of position (Supp. Figs. 3 and 4), the toad must rearrange the tongue and prey into proper position prior to reinitiating an HDA and swallow. Successful swallowing never occurs without an HDA motion, which indicates that movements of the hyoid apparatus are vital for intrabuccal prey manipulation and swallowing mechanisms.

Our results also highlight the importance of prey removal mechanisms for frogs with adhesive tongues. The soft tongue pad and sticky saliva are beneficial for prey capture (Pelli‐Martins et al. 2012; Kleinteich and Gorb 2014; Noel et al. 2017), and potentially intrabuccal prey manipulations (see Supp. Figs. 3 and 4) but may require specific prey removal mechanisms for swallowing to occur successfully. We observed that the prey remains adhered to the tongue tip at 50% through a feeding cycle, just prior to swallowing (ex., Video 5). Even if a toad repositions the tongue within the mouth during a double-swallow, the prey remains attached to the tongue throughout manipulation (see Video 3). Following the HDA, the hyoid plate is “scraped” against the roof of the toad's mouth as it moves anteriorly, which appears to function to mechanically remove the prey from the tongue (Videos 3 and 5). As the anterior part of the hyoid continues to move anteriorly, the posterior part of the hyoid is pulled dorsally, eventually positioned just under the junction between the tongue tip and the prey (Fig. 8). At this point, the prey is just anterior to the esophagus, ready for swallowing. The prey does not move into the esophagus until it is removed from the tongue tip by the rotation of the posterior part of the hyoid apparatus into its resting conformation (Fig. 8). This supports the hypothesis that prey must be mechanically removed from the tongue prior to swallowing (Kleinteich and Gorb 2014) and suggests that prey removal is one of the major functions of hyoid movements during prey transport. The scraping behavior of the hyoid may have implications for various structures located on the roof of the mouth in other frog species (see below for further discussion).

The swallowing phase involves movements that push prey past the pharynx and into the esophagus. Our results show that swallowing occurs when the posteromedial bones of the hyoid apparatus rotate back into resting position (Fig. 8; 50–60%). This movement coincides with prey being pinched off the tongue tip, presumably as the m. petrohyoideus anterior constricts the prey into the front of the esophagus. In *R. marina*, the m. petrohyoideus anterior has fibers that insert into the pharynx, the ventral part of posterior lobe of the alary process of the hyoid, and the dorsal surface of the hyoid plate near the midline (Ecker 1889; Trewavas 1932; see Fig. 9). This muscle is thought to function like the m. constrictor pharyngis in humans (Ecker 1889; Székely and Matesz 1993), which constricts around the food bolus to move it further into the esophagus. The prey is then pushed into the esophagus, concluding the swallow. Muscles of the tongue (e.g., the m. genioglossus) may also activate to pull the tongue anteriorly and free of the prey. In Video 5, previously ingested crickets move posteriorly in the alimentary canal as the most recent addition enters the esophagus, which allowed us to accurately estimate swallowing times.

In previous reports on amphibian feeding mechanics, it has been suggested that eye retraction plays an important role in swallowing prey, either by fixing the prey in place or pushing it backwards into the esophagus (Larsen and Guthrie 1975; Regal and Gans 1976; Deban and Wake 2000; Nishikawa 2000; Schwenk 2000; Levine et al. 2004; Noel et al. 2017; Witzmann et al. 2019). Frogs have large interpterygoid vacuities that allow the eyeball to move into the buccal cavity via action of the m. retractor bulbi, which we successfully simulated during our dissections of *R. marina*. However, our observations of *R. marina in vivo* indicate that depression of the eyes into the buccal cavity is unnecessary for swallowing. Our animals consistently closed their eyes during the prey capture phase, potentially for protection from moving prey (Nishikawa 2000; Levine et al. 2004), but the eyes were nearly always open when swallowing occurred (eyes open ∼28% through a feeding cycle, prior to onset of the HDA; Video 5, Table 1). We also observed that the site of swallowing occurs posterior to the back of the skull for small prey, well posterior to the interpterygoid vacuities (Video 5). It is thus unlikely that eye retraction plays a mechanical role in swallowing events of small prey in *R. marina*. However, because *R. marina* and many other frogs sometimes consume vertebrate prey (Paluh et al. 2021), it is possible that frogs use different mechanisms when feeding on prey that are large relative to the frog's oral cavity. Indeed, *R. marina* often close their eyes when swallowing large prey such as adult mice (R. Keeffe, pers. obs.). An important area for further study is how the mechanics of prey manipulation and swallowing vary with prey size and type.

### Pectoral girdle, skull, and jaw coordination during feeding

Throughout the first half of the feeding cycle, we find evidence for coordinated movements of the skull, jaw, and the pectoral girdle. These movements can be broken down into three phases: (1) preparatory aiming motions, (2) mouth opening motions, and (3) HDA motions. Preparatory motions of the skull and girdle vary depending on prey position, whereas motions that occur during mouth opening and the HDA are more consistent among our observed events. We discuss the functional implications of these motions in turn.

Our results corroborate previous work that concluded how positioning of the skull, jaw, and upper body during the preparatory phase were important to feeding success in *R. marina* (Ingle 1976; Gans and Gorniak 1982a). The “preparatory phase” of the feeding cycle is the period during which the toad lines up its head with the prey before the tongue is protruded from the mouth. We found that our individuals appeared to have little control of lateral movement of the tongue and a finite limit for tongue stretching, and thus coordinated movement of the forelimbs and head prior to tongue protrusion is paramount to a successful strike. Preparatory motions in frogs include radial turning of the upper body via the forelimbs, adjustment of head elevation, and occasionally lunging behaviors (Gans 1961; Ingle 1976; Gans and Gorniak 1982a; Deban and Nishikawa 1992; Wolff et al. 2004). We found that a toad rotates its upper body and head to align the midline of its skull with the prey before opening its mouth, although sometimes opening of the mouth occurs while swinging the head into position (Video 2). In some cases, we observed lunging behavior that correctly positioned the head for a successful feeding event (Supp. Fig. 7). Even when we did not observe lunging behavior, the toads always brace their arms against the substrate to mediate movements of the skull and upper body during the feeding cycle. This bracing motion likely causes the subtle movements of the pectoral girdle observed during this part of the feeding cycle (Fig. 10).

When the mouth is open, the movements of the skull and pectoral girdle have less variation between different feeding events than when the mouth is closed. The skull pitches dorsally and translates anterodorsally when the mouth opens. This likely helps control the trajectory of the tongue (Nishikawa 2000). We found that the movements of the jaw relative to the skull are tightly associated between successful events from the same individual (Fig. 12). From rest, the lower jaw rapidly rotates (< 0.11 ms) to about 40° during the period of tongue protrusion, pauses briefly, then just as rapidly opens another 30° to achieve maximum gape to begin tongue retraction, and then rapidly (< 13 ms) closes once the tongue tip in inside the buccal cavity (Fig. 12). In bufonids, tongue projection is facilitated by the rapid opening of the lower jaw through the elastic recoil of the m. depressor mandibulae (Nishikawa 2000; Mallett et al. 2001; Lappin et al. 2006). The pause we find between the initial jaw opening motion (which coincides with protrusion onset) and the subsequent opening motion (which coincides with retraction onset) may suggest that such elastic recoil may not only participate in tongue protrusion but also in tongue retraction mechanisms. We modeled the lower jaw as a single rigid body in our models, but we recognize that maximum gape may be facilitated by mandibular bending of the lower jaw, caused by contraction of the m. submentalis (Deban and Nishikawa 1992; Nishikawa 2000). We observed apparent bending of the lower jaw in many of our videos (e.g., Video 2). Exploring the relationship between mandibular bending, elastic recoil of the lower jaw, and function of the m. hyoglossus will be critical for developing a more complete model of tongue protrusion and tongue retraction mechanisms in the future.

Once the mouth closes and the HDA begins, the pitch of the skull returns to normal and the distance between the skull and the pectoral girdle reaches a maximum (Figs. 7 and 11). This dynamic displacement of the skull relative to the pectoral girdle may increase the area of the buccal cavity and thereby help facilitate the rotation of the hyoid apparatus that occurs during the HDA (Fig. 8). The motion is mechanically interesting because anurans lack a neck and generally have only eight or nine vertebrae that form a stiff spinal column (Nussbaum and Naylor 1982). Rotation of the skull likely occurs due to shortening of the epaxial musculature, such as the m. intertransversarius capitus superior and m. intercrurales, that are posterior to the skull. If true, then there are similarities between the feeding of *R. marina* and that of many fishes in which the epaxial muscles elevate the cranium during suction feeding (Camp et al. 2015).

The translations and rotations of the pectoral girdle are subtle and more difficult to observe than those of the skull. Unlike the movements of the skull that likely relate directly to tongue and hyoid movements, movements of the pectoral girdle are probably related to activity of postcranial muscles. We observed little translation of the pectoral girdle in most feeding events (Video 5; Fig. 10) despite activation of the m. sternohyoideus, located between the pectoral girdle and the hyoid apparatus, during prey transport (Gans and Gorniak 1982a). This implies that postcranial muscles (such as those between the pectoral girdle and the forelimbs) stabilize the pectoral girdle throughout the feeding cycle. The pectoral girdle might then act as an anchor during the feeding cycle for mobile elements like the hyoid apparatus and skull.

To our knowledge, our study is the first to quantify pectoral girdle movements during feeding, though its movement during locomotion is already documented (Emerson 1983, 1984). Anuran pectoral girdles are often characterized as being either arciferal or firmisternal. In firmisternal girdles, the clavicles and coracoids are fused at the midline, forming a rigid structure. In arciferal girdles, the clavicles and coracoids are not fused in the midline, united instead by cartilage and permitting the left and right pectoral girdles to move independently of one another. Emerson's (1983) experimental work on the anuran pectoral girdle found that the epicoracoid cartilages of the right and left halves of the pectoral girdle move ∼2 mm horizontally towards each other when landing from a jump, via compressive loading through the glenoids. She also found that such loading causes 10–13° of rotation at the anterior end of the girdle. These measurements align closely with those of our study, although because our toads are much larger (∼20 cm SVL) than those in Emerson's (1983) work (∼9 cm SVL), the deformation is proportionally smaller. These similarities suggest that deformation of an arciferal pectoral girdle not only functions to distribute stress when landing, but also during less dynamic events such as when a toad leans towards prey when feeding.

### Novel understanding from atypical feeding events

Through examination of “atypical” feeding events, we provide a basis for future work on the neurological correlation between different phases of the anuran feeding cycle. When a toad misses the prey, we observed that all kinematic steps proceed normally except that the HDA is omitted (Fig. 5B, Tables 1–3). We can draw two major conclusions from this. The first is that up until maximum retraction of the tongue, all tongue and hyoid retraction motions occur in both hits and misses and are likely obligate once a feeding event is initiated. Secondly, it suggests that *R. marina* can sense that a miss has occurred during the short period (136.44 ± 7.63 ms; Table 2) of tongue retraction and choose not to perform an HDA or a swallow (but still must proceed with the recovery phase). It might be that sensation of prey on the tongue pad is necessary for the toad to attempt an HDA and a subsequent swallow, although we did observe an event where the toad missed the prey and then performed a delayed swallow (Supp. Table 2).

Previous work has found that stimulation of the tongue elicits reflexive movement of the esophagus (Kajiya et al. 1993). Reflexive swallowing responses in *R. marina* would be a valuable direction of study, given our observation of diverse swallowing events. We observed toads to: (1) readjust prey within the mouth between mouth closure and swallowing (Video 4), (2) perform several HDA movements during the same event, (3) contact the prey with the tongue, fail to capture it, and then not swallow (Video 1), and (4) miss the prey but perform a delayed swallow (RM03). All of these suggest that swallowing reflexes are not a direct consequence of the tongue being stimulated by the presence of prey against it. The diversity of swallowing events suggest that toads have more control of prey movements within the mouth following mouth closure than would be expected if the entire process was a reflex. Whether certain movement patterns like those of the HDA and pharyngeal contractions are reflexive remains unknown and requires further examination.

Additionally, we observed that posteroventral translation of the hyoid apparatus always coincides with tongue retraction. This motion of the hyoid likely facilitates tongue retraction and positions it for further manipulation by the hyoid. We observed several cases of a double-swallow event where the toad readjusts the position of the tongue and prey by swinging the hyoid apparatus anteriorly following the offset of tongue retraction (Video 2). This pushes the tongue (and its attached prey) back into the front of the mouth for another swallowing event using the hyoid. There are several implications of this: (1) full protrusion of the tongue is not necessary for full retraction of the tongue; and (2) while maximum retraction of the tongue occurs obligately after a protrusion event, the toad can choose to either proceed with a swallow or reposition the tongue in the buccal cavity. While we did not collect data on neuromuscular relationships, such as EMG data, in this study, the obligate and facultative movements of the hyoid apparatus during prey transport warrant further investigation.

### Implications for anuran evolution

Our findings on the dynamic function of the hyoid apparatus during prey transport and swallowing lead to new interpretations of the morphological diversity of buccal anatomy observed across Anura. The hyoid skeleton varies widely across clades (Trewavas 1932; Duellman and Trueb 1986), but there have been few functional interpretations of this variation other than speculation related to suction feeding (although see Nishikawa 2000). We propose that our findings of the movements of the hyoid apparatus during prey transport and swallowing open new lines of inquiry into the function of both hyoid diversity and enigmatic features of the buccal cavity.

#### Structures of the buccal roof

Our observations of hyoid movement provide context for variation in the buccal roof anatomy in frogs. During the prey transport phase, prey is removed from the sticky tongue by the hyoid scraping against the roof of the mouth. Many frogs possess small vomerine teeth on the palate (Paluh et al. 2021) that are anterior to the interpterygoid vacuities of the eyes. Regal and Gans (1976) proposed that the vomerine teeth play a role in the movement of prey in the mouth via the tongue, even potentially grazing the prey against those teeth to help with taste discrimination. These teeth may also play a role in removing prey from the tongue via hyoid movement. *Rhinella marina* does not have vomerine or any other teeth, and the substrate of the HDA appears to be the lateral bar formed by the pterygoid and parasphenoid bones.

In other edentulous frogs, there are additional structures in this area of the mouth. Within the Microhylidae, many genera have what are termed “pharyngeal folds” that occur across the palate in front of the pharynx. These consist of transverse dermal folds with the posterior-most fold being stiff and denticulate, those anterior usually less so. These folds are diagnostic of the Microhylidae (Parker 1934; Zweifel 1972) and not known in other frogs, but they have no known functional significance. If microhylids have similar hyoid motions to *R. marina*, these folds may serve to help remove prey from the tongue prior to swallowing, especially in species with rapid tongue projection. The pipoid *Rhinophrynus dorsalis* also has “mesa-like epithelia projections” that overlap with deep, parallel grooves in this same area of the buccal cavity (Trueb and Gans 1983). These structures may help to protect the frog from the bites of its specific prey (Trueb and Gans 1983), termites, but they might also play a role in removing prey from the tongue. Deeper examination of the presence and diversity of these structures may help us to better understand differences among frog feeding ecologies and swallowing mechanics.

#### Hyoid morphological diversity

Variation of the hyoid skeleton across frogs may provide insight into how swallowing movements vary among species. The typical anuran hyoid consists of a central hyoid plate from which three pairs of cartilaginous processes (from anterior to posterior: hyale, anterolateral, posterolateral) and one pair of ossified processes (posteromedial) project (Fig. 2). The hyoid plate is usually cartilaginous and varies widely in surface area (Trewavas 1932; Duellman and Trueb 1986), and in some cases contains ossifications (ex. *Leiopelma*, *Rhinophrynus*, *Bombina*, *Crossodactylus*). The processes of the hyoid plate also vary. The hyalia can be ossified (ex. *Hymenochirus*), absent (ex. *Pipa*, *Xenophrys*), disjointed (ex. *Rhinophrynus*, *Pelodytes*), or can bear an additional anterior process (ex. *Atelopus*, *Heterixalus*, *Rana*, *Rhacophorus*), fenestra (ex. *Pseudopaludicola*, *Breviceps*), or a broad, anterior flange (ex. *Hemisus*, *Breviceps*, *Glyphoglossus*) (Ridewood 1897; Trewavas 1932; Trueb and Gans 1983; Cannatella and Trueb 1988a; see Fig. 2). Between the hyalia, there is a U-shaped gap termed the hyoglossal sinus through which the m. hyoglossus passes. The shape of this sinus varies from narrow to wide. The anterolateral and posterolateral processes of the hyoid plate vary in shape and length and are sometimes absent. In several early-diverging lineages of frogs, the hyoid apparatus includes an additional, V-shaped ossification ventral to the hyoid plate termed the parahyoid bone (Cannatella 1985). This feature has also been observed in extinct anurans that have been allied to these extant families (e.g., *Liaobatrachus*; Dong et al. 2013). Hyoid cartilages can be sexually dimorphic in some species, as well as asymmetrical in some individuals (Trewavas 1932); some individual variation was evident in the *R. marina* of our study (R. Keeffe, pers. obs). In addition to the skeletal structure of the hyoid, the musculature that attaches to the hyoid plate and within the tongue also varies (Horton 1982; Nishikawa 2000). There are three major types of tongue protrusion, each with different muscular configurations: mechanical pulling (ex. *Hyla*, *Ascaphus*, *Pelobates*), inertial elongation (ex., *Rhinella*, *Physalaemus*), and hydrostatic elongation (ex. *Hemisus*, *Dyscophus*) (Nishikawa and Cannatella 1991; Nishikawa 2000). The morphology of the hyoid apparatus correlates with specific configurations of muscular attachments and the degree of motion of which the hyoid is capable, which likely have kinematic consequences for prey transport and swallowing motions.

Caution is required in generalizing from the present study to the exceptional diversity of the hyoid apparatus and its attached musculature (see Figs. 2 and 9). We observed dynamic movements of the hyoid apparatus in *R. marina*, especially lateral motions of the hyalia and dorsoventral flexion of the hyoid plate during prey manipulation and swallowing. But these movements may not be applicable to all frog clades. For example, species with a relatively short hyoid plate may be unable to perform such complex flexions during the HDA (Fig. 2B). Similarly, species with short hyalia and anterior processes may be limited in the range of hyoid translations possible within the buccal cavity (Fig. 2D). Species with ossifications of the plate presumably have a stiffer hyoid that may not bend as easily as we observed in *R. marina* (Fig. 2F). Additionally, bufonids stand out among frogs by (1) having a hyoid plate that is much longer than it is wide, (2) lacking the anterior process of the hyalia, (3) lacking the m. petrohyoideus posterior secundus, (4) lacking the posterior part of the m. depressor mandibulae, (5) having the m. hyoglossus insert on the dorsal surface of the posteromedial process rather than on the ventral surface, and (6) lacking several major cartilages of the larynx (Figs. 9 and 15; Trewavas 1932; Griffiths 1963; Chacko 1965). At this point, we do not know which of these distinctive features have direct consequences for the feeding mechanics of *R. marina*, if any. The highly protrusible tongue of *R. marina* is likely not the plesiomorphic condition for anurans (Deban and Nishikawa 1990, 1992) with only a few anurans using ballistic tongue protrusion during feeding (Nishikawa 2000). In an intriguing similarity, the ballistic tongues that evolved twice independently in plethodontid salamanders were also associated with loss or proportional changes of musculoskeletal elements (Lombard and Wake 1986; Wake et al. 2015).

Two specific cases of hyoid morphotypes that almost certainly have different hyoid mechanics than those of *R. marina* are those of fossorial and aquatic specialists. For example, *Hemisus marmoratus*, a fossorial species with hydrostatic elongation of the tongue, has a robust hyoid apparatus with right and left hyalia that are bound together by a broad anterior flange (Trewavas 1932; Fig. 2C). This likely precludes the dynamic motions of the hyalia that we observed. *Hemisus* is unique among frogs for having a prehensile tongue, an ability tied to the function of the m. hyoglossus. When the m. hyoglossus is denervated, the tongue is no longer prehensile and the frog's swallowing ability is greatly hindered (Ritter and Nishikawa 1995, Tso et al. 1995; Nishikawa 2000). This might suggest that the HDA would not be necessary for swallowing prey in *Hemisus* because its tongue could perform all necessary prey manipulation motions. *Rhinophrynus dorsalis* is another fossorial specialist with a bizarre hyoid (Fig. 2F). Its hyalia are segmented, separated by a wide hyoglossal sinus; moreover, the hyoid plate includes a long, thin parahyoid bone, and the plate itself is short (Trueb and Gans 1983). This morphology may stiffen the hyoid plate and prevent the sliding that we observed in *R. marina*. The musculature associated with the hyoid in *Rhinophrynus* also likely prevents some of the movements that we observed. *Rhinophrynus* lacks the m. omohyoideus, its m. geniohyoideus is single-lobed rather than double-lobed, and its mm. petrohyoideus attaches dorsally to the hyoid plate rather than ventrally. Previous authors suggested that tongue protrusion in *Rhinophrynus* incorporates anterior movements of the hyoid to help extend the tongue (Trueb and Cannatella 1982; Trueb and Gans 1983), which we did not observe in *R. marina*.

Anurans that feed aquatically often lack or have a reduced tongue (Fabrezi and Lobo 2009; Barrionuevo 2016), and at least some feed via suction (Carreño and Nishikawa 2010; Barrionuevo 2016; Cundall et al. 2017). The most extreme of these is *Pipa pipa* (Fig. 2E), a tongueless suction-feeder with a massive hyoid that is not attached to the skull. The hyoid is retracted by the mm. rectus abdominis profundus, which inserts on the femur, rather than by the m. sternohyoideus, which inserts on the pectoral girdle as in most frogs (Cundall et al. 2017). Other pipids with suction-feeding also have massive and heavily ossified hyoid skeletons (Sokol 1969; Cannatella and Trueb 1988b; Carreño and Nishikawa 2010). The most well-studied non-pipid with suction-feeding is *Telmatobius rubigo*, which has a robust m. sternohyoideus that helps retract the hyoid quickly to create suction for feeding underwater (Barrionuevo 2016). The hyoid plate of *Telmatobius* is short and partially mineralized, which probably prevents the bending that we observed in *R. marina*. Barrionuevo (2016) noted a connection between tongue reduction and hyoid ossification in aquatic frogs, suggesting that hyoid rigidity is important for suction feeders. Ossifications in *T. rubigo* occur near the attachment site of m. sternohyoideus, suggesting those areas are under high loading stress during suction.

Aquatic and fossorial anurans are examples of the extremes of feeding diversity among frogs. However, hyoid morphology and buccal musculature vary widely among frog families that lack specialized feeding modes like hydrostatic tongues and suction-feeding. Bufonids are edentulous and primarily capture prey using the tongue, but many frogs use a combination of tongue prehension and jaw prehension (aided in many species by maxillary teeth and odontoid fangs; Paluh et al. 2021) that should be considered in future investigations of anuran feeding mechanics (Monroy and Nishikawa 2011). Frogs are widely considered to be gape-limited predators with little discrimination between prey types (Regal and Gans 1976). Yet the diversity of hyoid morphology observed across frogs may indicate a diversity of ways in which frogs process and swallow prey of different sizes and types. Hyoid diversity is likely not shaped only by feeding and swallowing, but also by respiration and vocalization. Fruitful directions for research include understanding the tradeoffs in hyoid anatomy and movement between different functional demands as well as their relationship to different diets and diversification of various frog lineages.

## Conclusion

Our work is the first to show the extensive movements of the tongue and hyoid during swallowing in frogs. We show that prey capture is only a small portion of the total feeding cycle with prey transport, swallowing, and recovery comprising most of the cycle. We find remarkable retraction of the tongue, which consistently stretches posterior to the skull during prey transport, often more than it is stretched during maximal protrusion. We also found that most of the hyoid's movements during the feeding cycle occur during prey transport and swallowing, where it appears to play a role in the removal of prey from the tongue. Feeding kinematics are largely similar among the three individuals studied as well as between hits and misses. One source of variation recovered among feeding trials was the distance of the prey, which alters the timing of some events in the feeding cycle. The pectoral girdle moves little during feeding, which may indicate that postcranial muscles actively stabilize the girdle during feeding.

The movements of the hyoid that we observed in *R.marina* suggest new directions for studies of functional morphology in frog feeding. There are nearly 7500 species of frogs and many lineages have unique hyoid and tongue morphologies. A larger, comparative study incorporating representatives with different feeding modes would deepen our understanding of the evolution and diversity of feeding mechanics in frogs. Another valuable direction is a closer examination of the neuromuscular relationship between tongue protrusion and swallowing in frogs. Our few recordings of misses suggest that some movements of prey transport are obligate, while others are facultative. Our findings show that cane toads always fully retract the tongue after tongue protrusion even during a miss, which is different from what is seen in other tetrapods (Kier and Smith 1985). This may indicate a unique neuromuscular association and have implications for the evolution of feeding in frogs.

## Supplementary Material

obac045_Supplemental_Files

## Data Availability

The data that support the findings of this study are openly available at the XMA Portal repository (https://xmaportal.org/) as part of the electronic supplementary material for this study.
