# Supplementary material for: XROMM Analysis of Feeding Mechanics in Toads: Interactions of the Tongue, Hyoid, and Pectoral Girdle
Source: Integr Org Biol. 2022 Nov 15;4(1):obac045. doi: 10.1093/iob/obac045 (PMC9665897; doi:10.1093/iob/obac045)
Supplement: obac045_Supplemental_Files [file obac045_supplemental_files.zip › Spanish_Abstract.docx]

Durante la alimentación en muchos vertebrados terrestres, la lengua actua en combinación con el hioides y la cintura ó faja pectoral. En las ranas, estos tres elementos estan conectados con músculos. Aunque la mecánica de la alimentación de la lengua de los anuros esta bien estudiada, se sabe poco acerca de como el movimiento de la lengua se relaciona con los movimientos del esqueleto or como se mueven as estructuras bucales despues de que se cierra la boca. Mientras que algunas características como la cintura pectoral y el hioides no se ven externamente en las ranas, sus movientos se pueden seguir usando video de rayos X. Usamos técnicas de XROMM (reconstrucción de rayos x de morfología en movimiento) para seguir los movimientos de la lengua, el hyodeo, la cintura pectoral, cráneo y mandibula en 3D, durante el ciclo de alimentación del sapo de caña <i>Rhinella marina</i>. Mostramos que los movimientos de estos elementos estan integrados durante la protrusión de la lengua y la captura de la presa, ademas de durante el transporte de la presa, tragado y recuperación. Nuestros resultados demuestran que el hioides es importante para manipular la presa y para tragar. La lengua se estira posterior al cráneo durante la fase de tragar, muchas veces aún más que el estiramiento que presenta cuando atrapa la presa. La cinemática de la alimentación es similar entre individuos y la cinemática de intentos fallidos es similar a la de los intentos exitosos. Nuestros datos proven una nueva perspectiva del rol potential en la alimentación de la cintura pectoral, un element que se usa primordialmente en la locomoción. Este trabajo sugiere nuevas preguntas acerca de la evolución de la alimentación en las ranas, así como de la influencia de la diversidad de los pectorales y la anatomía buccal que se observan en los anuros en la cinemática de alimentación.
