# Supplementary figures and images for: XROMM Analysis of Feeding Mechanics in Toads: Interactions of the Tongue, Hyoid, and Pectoral Girdle
Source: Integr Org Biol. 2022 Nov 15;4(1):obac045. doi: 10.1093/iob/obac045 (PMC9665897; doi:10.1093/iob/obac045)

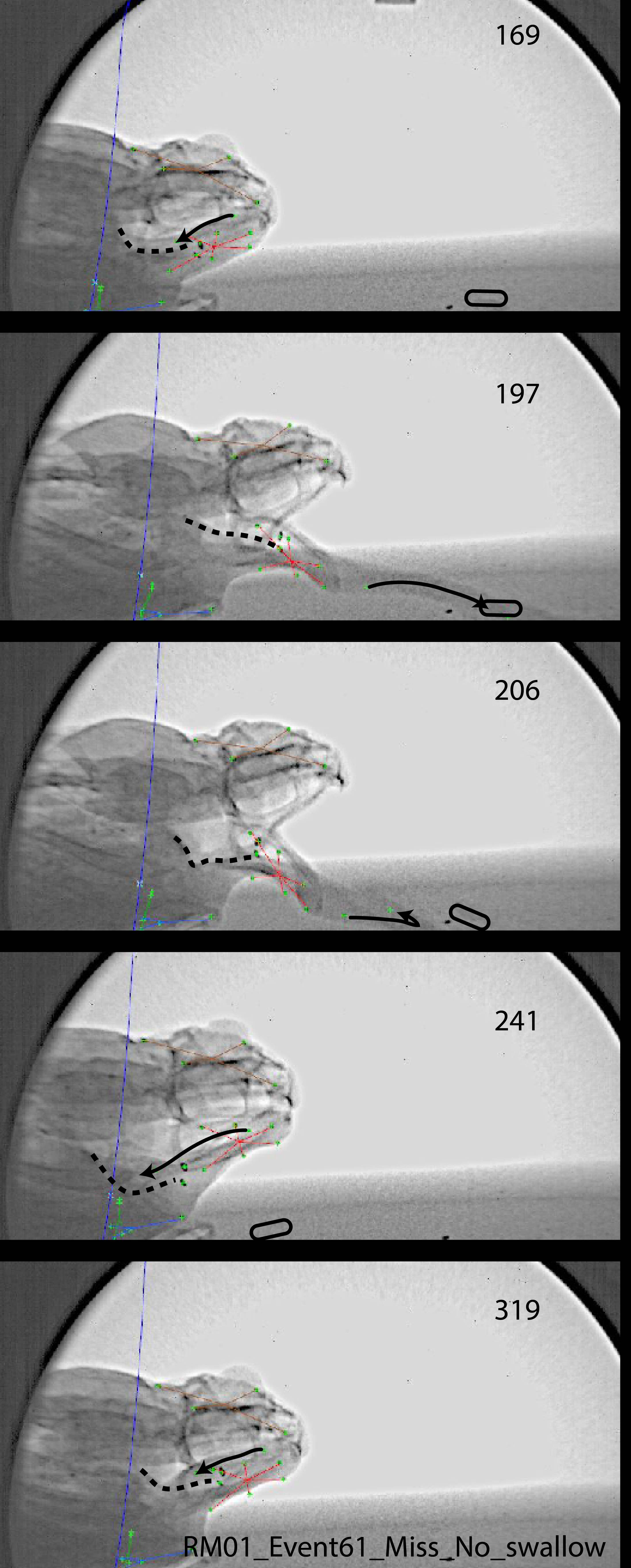

Supplement: obac045_Supplemental_Files [file obac045_supplemental_files.zip › Supplemental_Figure_1.tif]

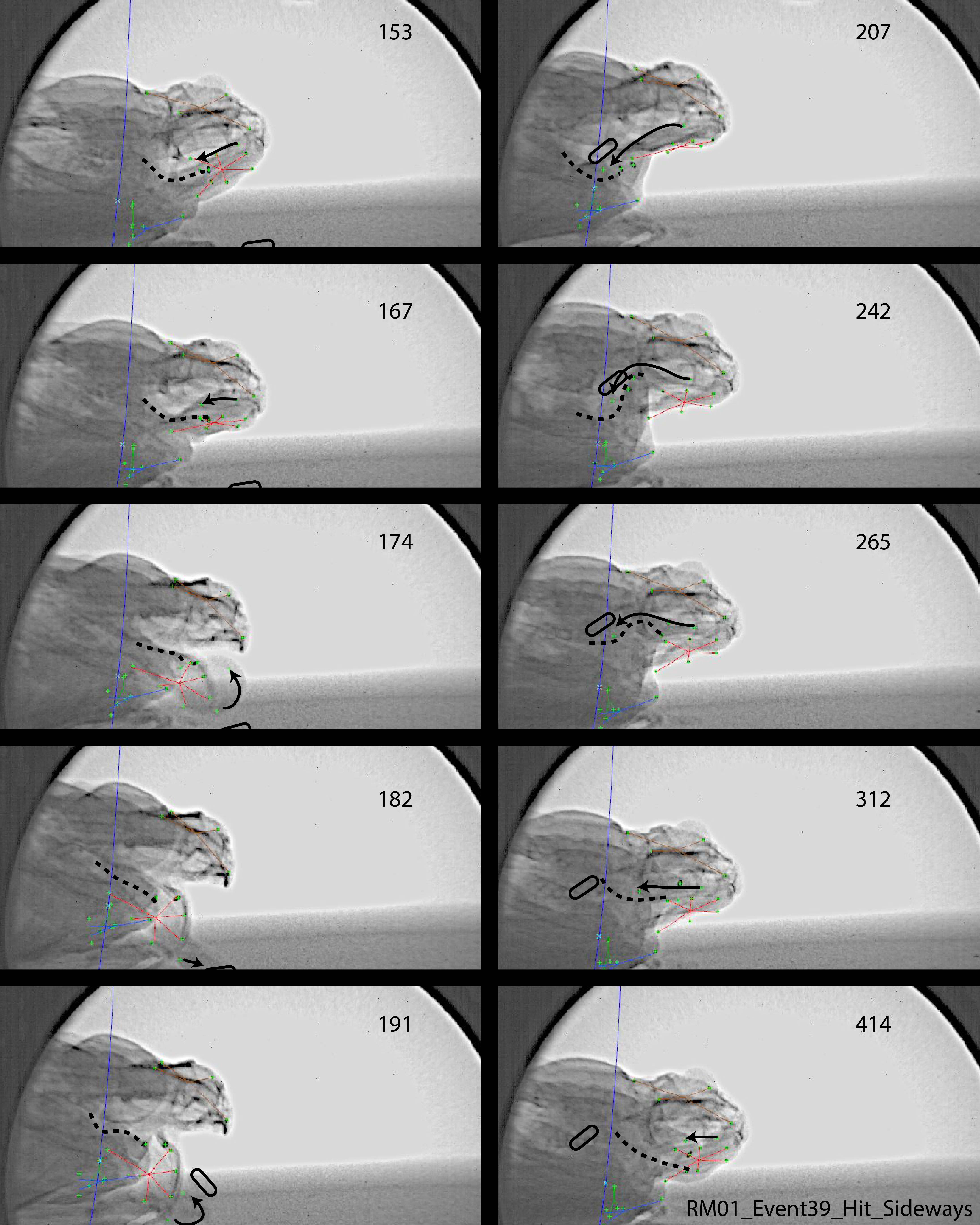

Supplement: obac045_Supplemental_Files [file obac045_supplemental_files.zip › Supplemental_Figure_2.tif]

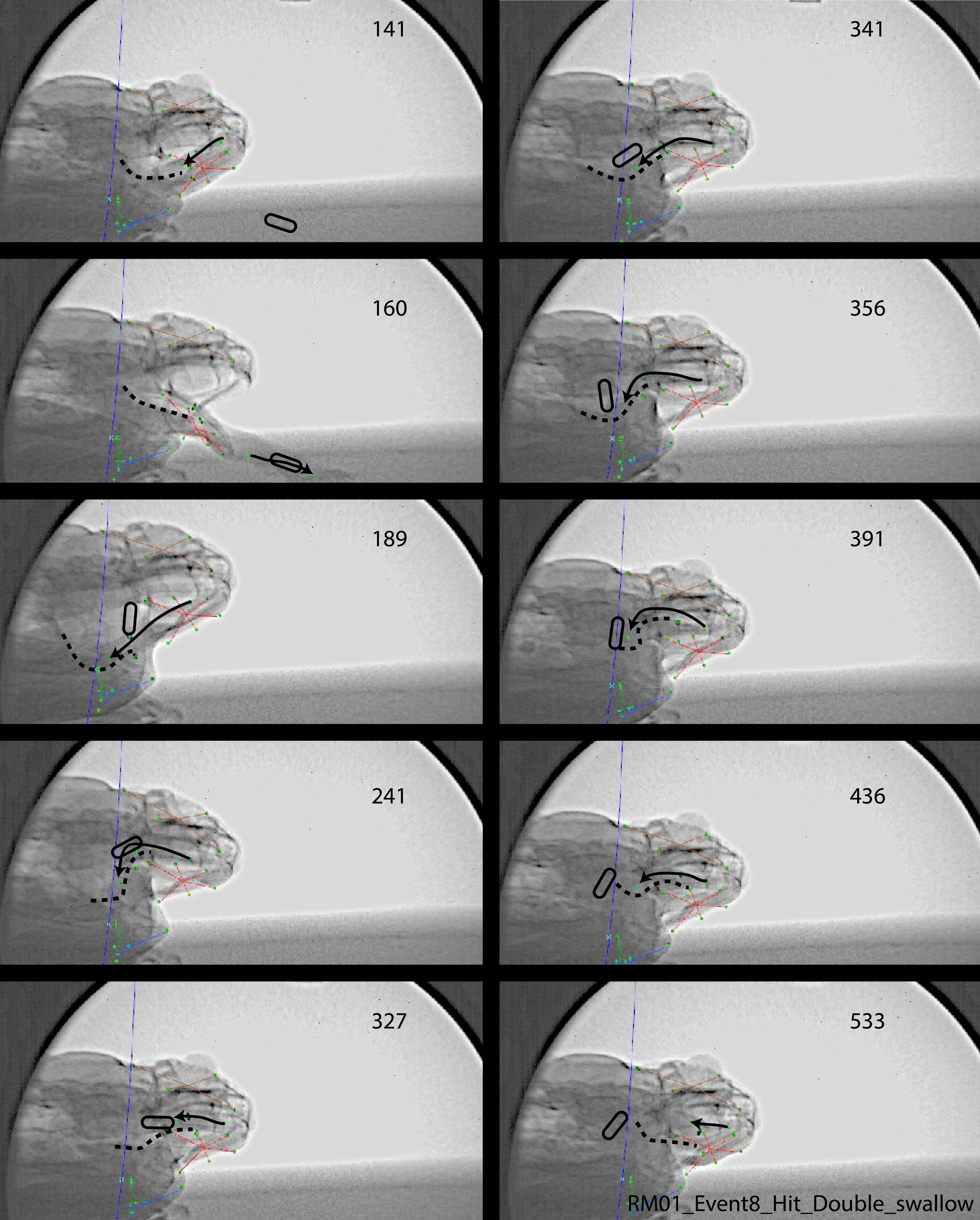

Supplement: obac045_Supplemental_Files [file obac045_supplemental_files.zip › Supplemental_Figure_3.tif]

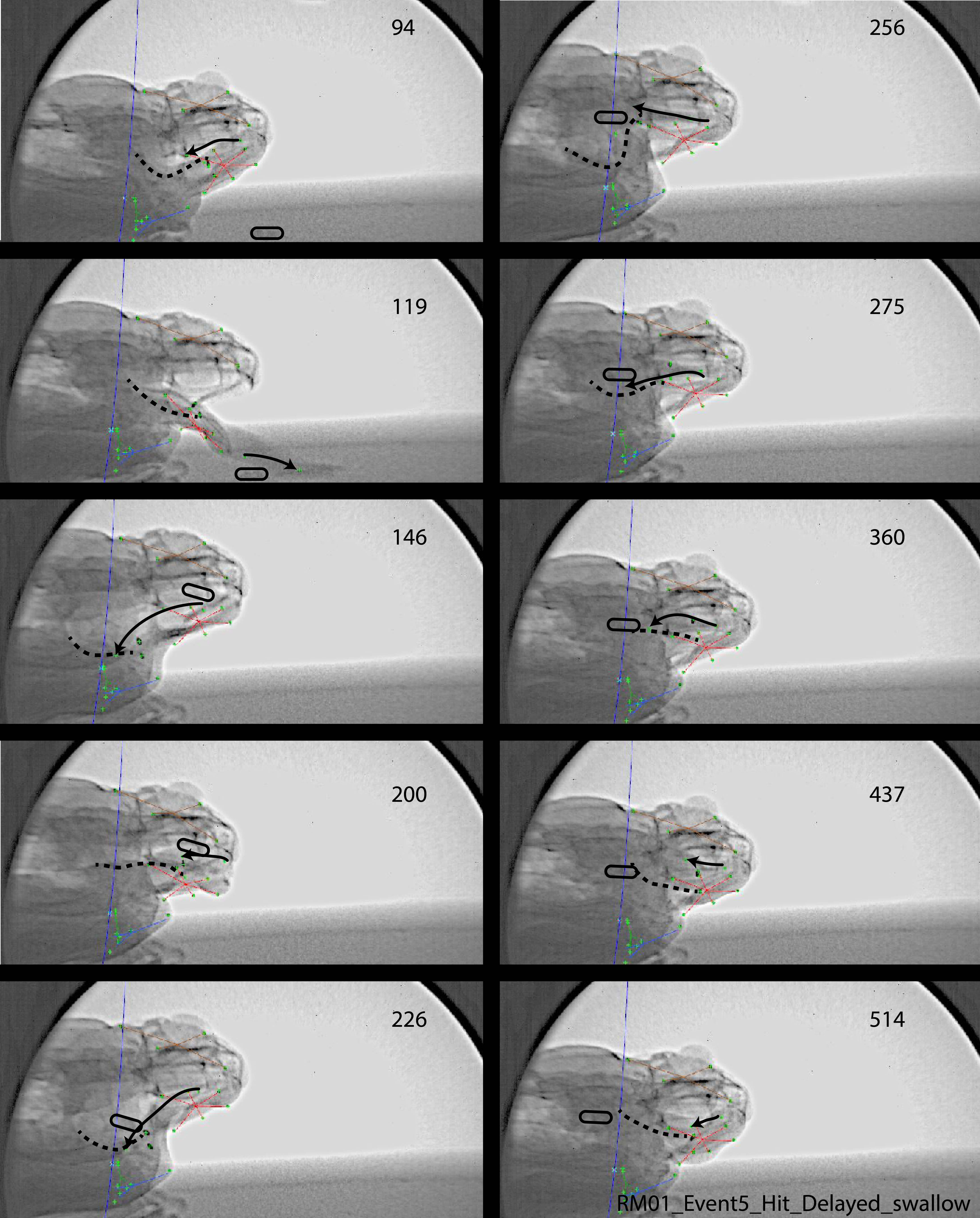

Supplement: obac045_Supplemental_Files [file obac045_supplemental_files.zip › Supplemental_Figure_4.tif]

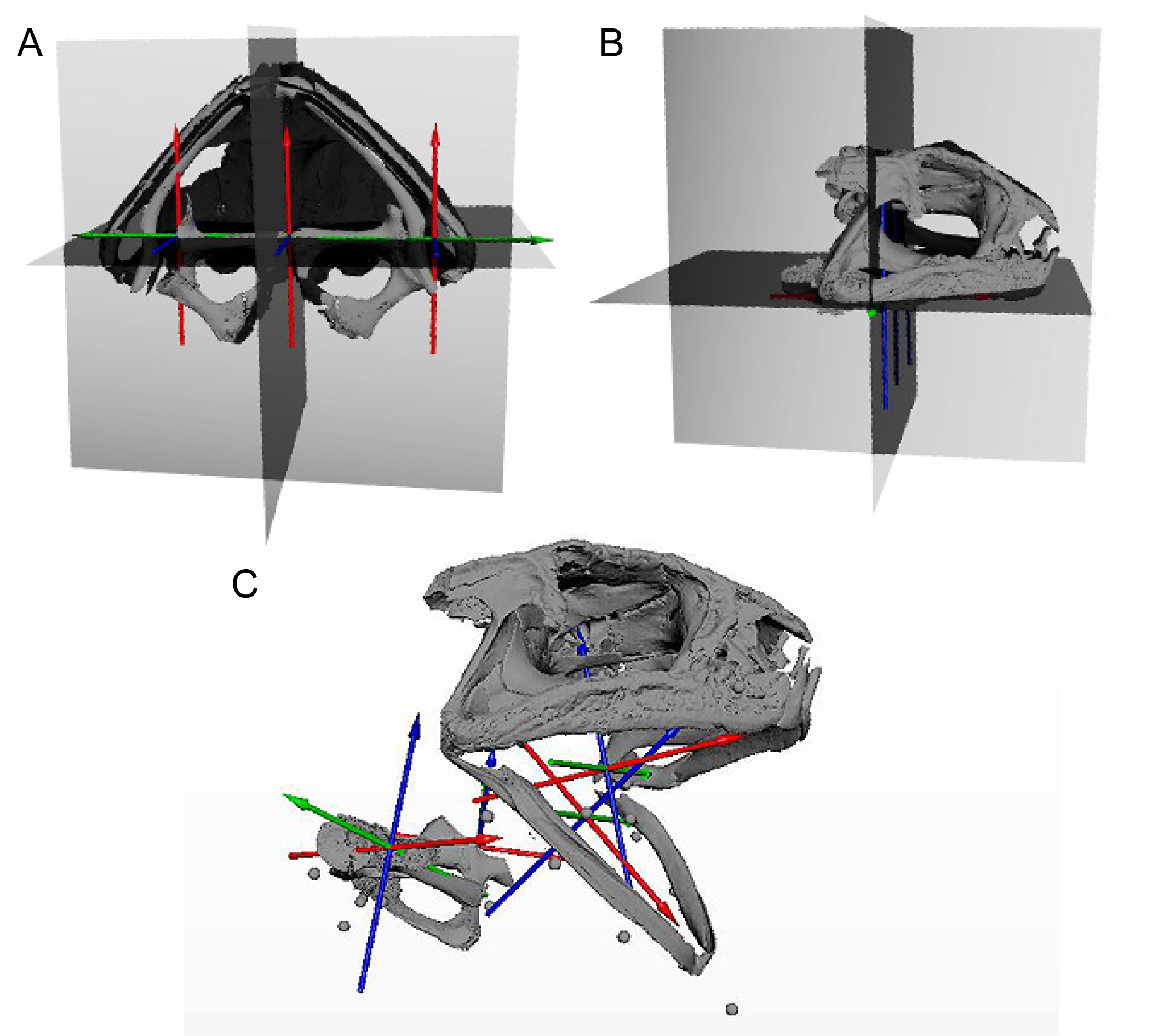

Supplement: obac045_Supplemental_Files [file obac045_supplemental_files.zip › Supplemental_Figure_5.tif]

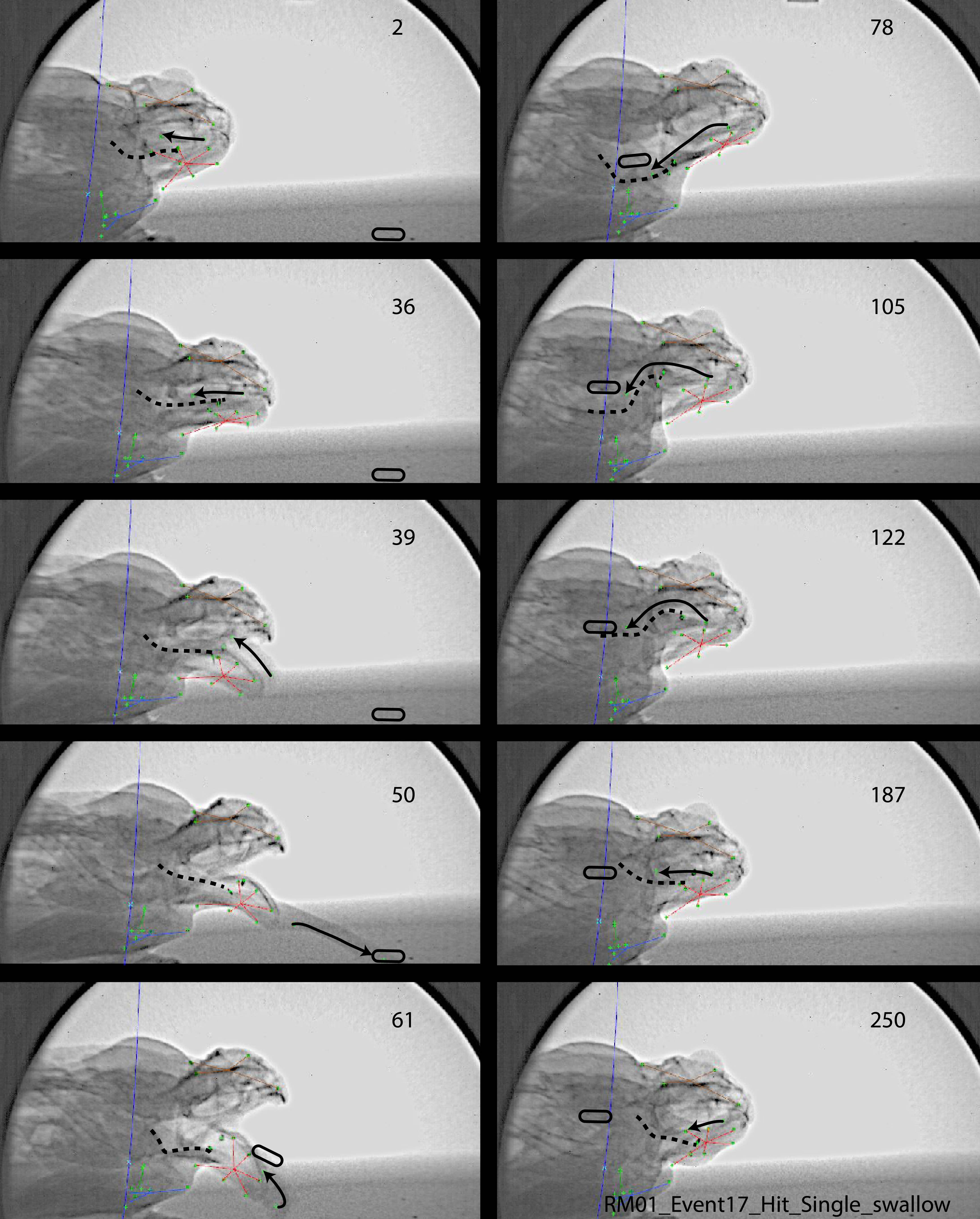

Supplement: obac045_Supplemental_Files [file obac045_supplemental_files.zip › Supplemental_Figure_6.tif]

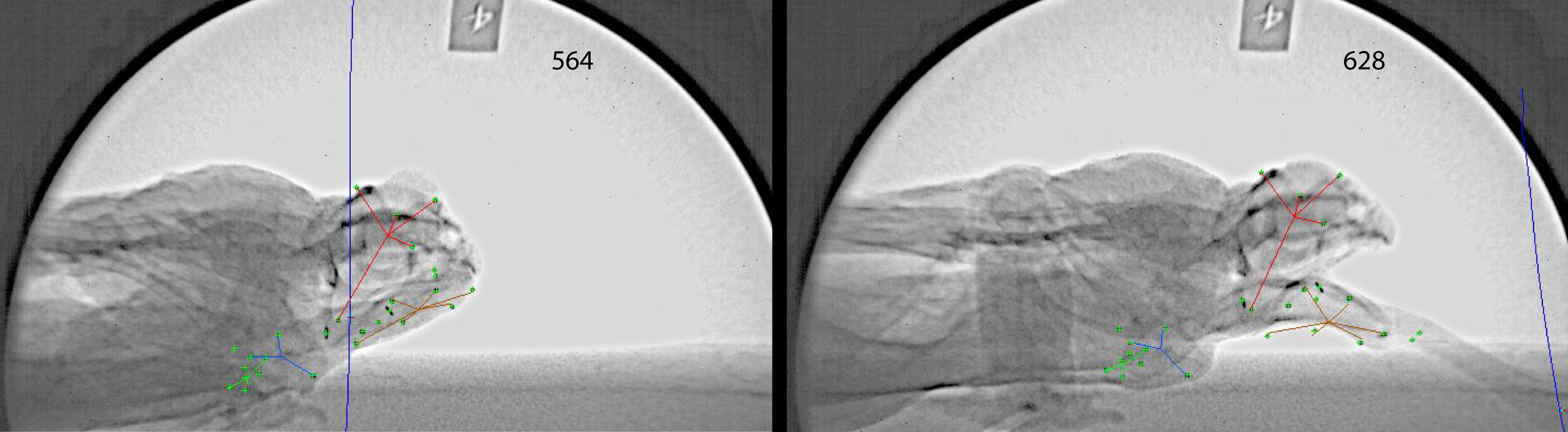

Supplement: obac045_Supplemental_Files [file obac045_supplemental_files.zip › Supplemental_Figure_7.tif]
